# Comprehensive genotyping of a Brazilian cassava (*Manihot esculenta* Crantz) germplasm bank: insights into diversification and domestication

**DOI:** 10.1007/s00122-021-03775-5

**Published:** 2021-02-11

**Authors:** Alex C. Ogbonna, Luciano Rogerio Braatz de Andrade, Lukas A. Mueller, Eder Jorge de Oliveira, Guillaume J. Bauchet

**Affiliations:** 1 Cornell University, Ithaca, NY USA; 2 Boyce Thompson Institute for Plant Research, Ithaca, NY USA; 3 Embrapa Mandioca E Fruticultura, Cruz de Almas, BA Brazil

## Abstract

**Key message:**

Brazilian cassava diversity was characterized through population genetics and clustering approaches, highlighting contrasted genetic groups and spatial genetic differentiation.

**Abstract:**

Cassava (*Manihot esculenta* Crantz) is a major staple root crop of the tropics, originating from the Amazonian region. In this study, 3354 cassava landraces and modern breeding lines from the Embrapa Cassava Germplasm Bank (CGB) were characterized. All individuals were subjected to genotyping-by-sequencing (GBS), identifying 27,045 single-nucleotide polymorphisms (SNPs). Identity-by-state and population structure analyses revealed a unique set of 1536 individuals and 10 distinct genetic groups with heterogeneous linkage disequilibrium (LD). On this basis, a density of 1300–4700 SNP markers were selected for large-effect quantitative trait loci (QTL) detection. Identified genetic groups were further characterized for population genetics parameters including minor allele frequency (MAF), observed heterozygosity $({H}_{o})$, effective population size estimate $\widehat{{(N}_{e}}$) and polymorphism information content (PIC). Selection footprints and introgressions of *M. glaziovii* were detected*.* Spatial population structure analysis revealed five ancestral populations related to distinct Brazilian ecoregions. Estimation of historical relationships among identified populations suggests an early population split from Amazonian to Atlantic forest and Caatinga ecoregions and active gene flows. This study provides a thorough genetic characterization of ex situ germplasm resources from cassava’s center of origin, South America, with results shedding light on Brazilian cassava characteristics and its biogeographical landscape. These findings support and facilitate the use of genetic resources in modern breeding programs including implementation of association mapping and genomic selection strategies.

**Supplementary Information:**

The online version of this article (10.1007/s00122-021-03775-5) contains supplementary material, which is available to authorized users.

## Introduction

Cassava (*Manihot esculenta* ssp*. esculenta*) domestication resulted from human-mediated selection in the Amazonian region and adjacent areas of northern Bolivia (Clement et al. 2010), initially in the early Holocene period (Lombardo et al. 2020), with significant changes in root size. Evidence suggests that cultivated cassava was domesticated once from *M. esculenta* subsp. *flabellifolia* (Olsen and Schaal 1999; Olsen 2004; Schaal et al. 2006). This hypothesis was further supported by Léotard and colleagues studying the G3pdh gene diversity among six wild *Manihot* species (Léotard et al. 2009). Domesticated cassava has been further separated into two groups, sweet and bitter cassava, based on root cyanogenic potential (Elias et al. 2004; Clement et al. 2010). Sweet cassava is generally cultivated throughout the Neotropics, but dominates the western and southern water-heads of the Amazon river basin; the bitter cassava type dominates Amazon’s northern water-heads and central portion where it is predominantly used for starch extraction and cassava flour production (Nordenskiold 1924; Renvoize 1972; Fraser et al. 2012; Mühlen et al. 2019; Ogbonna et al. 2020). Both types of cassava are valued for their starchy storage roots, especially by smallholder farmers.

Cassava breeding activities over the last few decades have made progress compared to other underutilized crops (Johnson et al. 2002; Kawano 2003; Okechukwu and Dixon 2008; Kittupadakul et al. 2017; Eriksson et al. 2018), although not comparable to its potential (Ceballos et al. 2004). Many challenges confronting cassava breeding include the use of heterozygous progenitors, long breeding cycles, clonal propagation and non-recovery of the recurrent genome after trait introgression (Ceballos et al. 2016; Kuon et al. 2019). While cassava is mostly clonally propagated, it is an outcrosser with plants still capable of sexual reproduction, thereby increasing allelic variation (McKey et al. 2010). Modern breeding activities have leveraged from its outcrossing capacity, notably in Africa with the development of interspecific crosses with *M. glaziovii*, “the rubber cassava,” leading to genomic introgressions, for key plant architecture traits (Nichols 1947; Lefévre and Charrier 1993; Prochnik et al 2012; Wolfe et al. 2019). However, the presence of introgressed segments from past breeding activities has not been explored in Brazilian germplasm. More widely, assessing genetic diversity from Brazilian germplasm collections and wild relatives would provide an allelic resource for additional adaptive, quality and reproductive traits to breeding populations. Therefore, the genotypic evaluation of the National Agriculture Research Institute of Brazil (Embrapa) cassava germplasm collection, covering 26 Brazilian states and five ecoregions, is an opportunity to identify potential population structures and related evolutionary events. So far, in situ cassava genetic diversity studies using smaller datasets and low density genotyping have reported population structure to be different across bitter and sweet Brazilian cassava, owing to high haplotype diversity and environmental heterogeneity of cassava-growing regions (Miller and Schaal 2005; Alves-Pereira et al. 2018). However, ex situ germplasm collections have revealed comparable structure and spatial genetic patterns (Oliveira et al. 2014; Albuquerque et al. 2018). Linking such agro-ecological information to available germplasm is essential in pre-breeding activities such as germplasm characterization and subsequent trait genetic architecture dissection (Dwivedi et al. 2016). Germplasm management and tracking is often challenged by its maintenance cost, the renaming of germplasm by farmers and breeders and a continuous increase in germplasm exchange globally (Kilian and Graner 2012). Therefore, in many situations breeding programs use only part of the existing germplasm (e.g., only clones that have already been well characterized and have high breeding value). However, complete knowledge of the genetic variability and other useful parameters for application in conventional or molecular breeding are essential for their rational use of the germplasm as well as for optimizing the genetic gains.

In addition, recent and rapid advances in molecular tools for indirect selection have brought progressive and continuous gains in plant breeding, such as genome-wide association studies (GWAS) and genomic selection (GS). In general, the success of GWAS relies on linkage disequilibrium (LD) that refers to allelic association and linkage of genetic markers in the genome. Evaluating and assessing the diversity and population genetic parameters, such as LD, of cassava germplasm in Brazil could help to address significant germplasm management challenges. Average LD decay defines the required number of SNP markers and mapping resolution in association studies (Flint-Garcia et al. 2003). LD decay and population structure have been extensively explored in sexually propagated plants including *Arabidopsis thaliana* and maize (Nordborg et al. 2002; Kim et al. 2007), but only recently in clonally propagated crops including potato, sweet potato and African cassava (Stich et al. 2013; Vos et al. 2017; Wadl et al. 2018; Rabbi et al. 2017; Ramu et al. 2017; Ferguson et al. 2019).

This study provides a genome-wide assessment of population structure, genetic diversity, linkage disequilibrium, introgressions, population splits and gene flow events from Brazil’s Embrapa Cassava Germplasm Bank. Taken together, the results will enable a more effective use of germplasm resources for various breeding-related activities, such as marker development, parents selection and gene mapping.

## Materials and methods

### Plant material

**Fig. 1:**
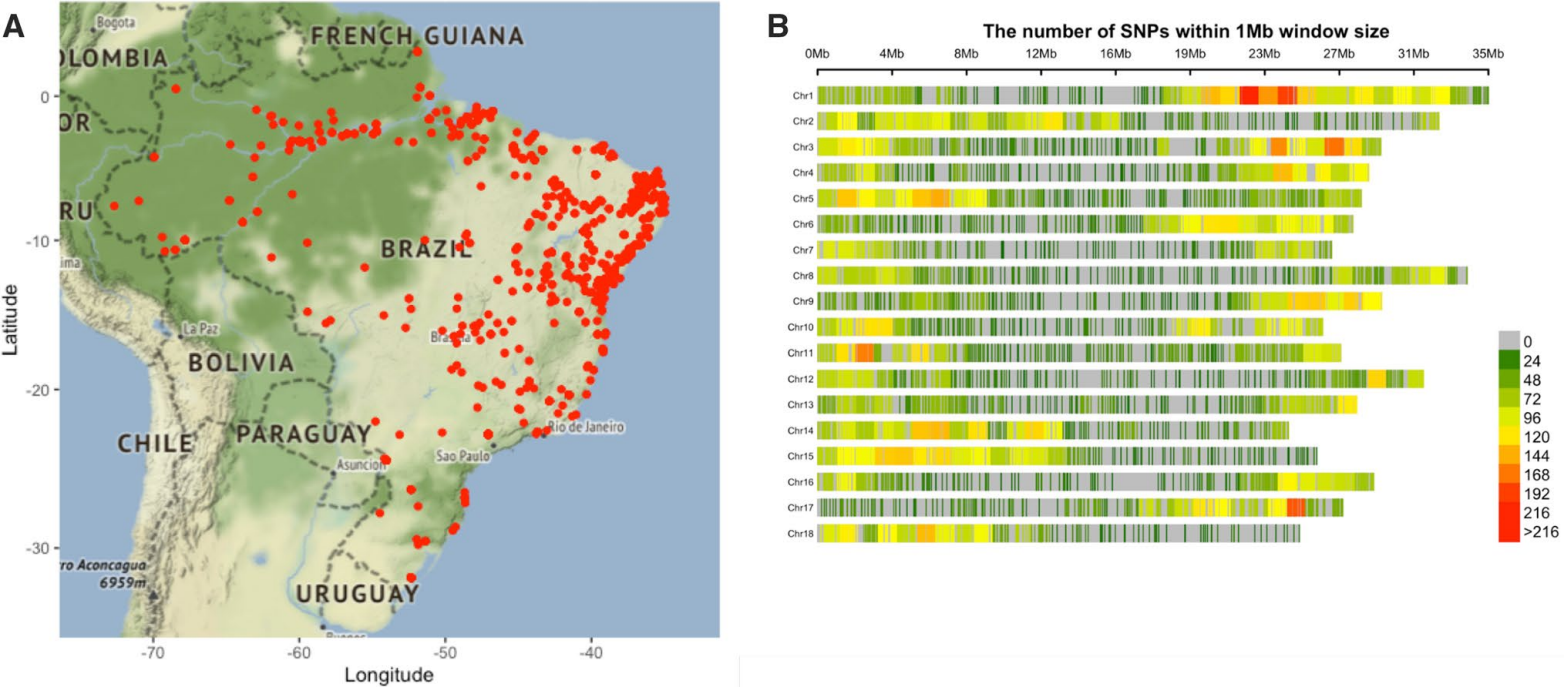
Germplasm geographic sampling and SNP distribution. **a** Geographic locations for cassava clones included in the germplasm collection. A total of 1821 cassava accessions have valid geographic information; each red dot shows the specific site in Brazil where an accession was sampled from. **b** Genotyping-by-sequencing single-nucleotide polymorphism (SNP) density in the Brazilian germplasm collection. The legend scale indicates the number of SNPs within 1 Mb window size. The plot shows the distribution of SNPs across the genome in our population

A total of 3354 accessions from the Cassava Germplasm Bank (CGB) of Brazilian Agricultural Research Corporation, Embrapa Mandioca e Fruticultura, located in Cruz das Almas, Bahia, Brazil (12 400 19″S, 39 060 22″W, 226 m altitude) were used for this study (Fig. 1a). The germplasm collection includes cassava landraces and modern breeding lines from various growing regions and ecoregions of Brazil. A subset (1580) of these 3354 accessions was previously described in Albuquerque et al. (2018). The years that each individual was collected ranged from 1962 to 2018, including a few accessions originally from Colombia (63), Nigeria (11), Venezuela (4), Uganda (2), Panama and Thailand (1). An additional set of 62 South American accessions from the cassava HapMap efforts were also included (Bredeson et al. 2016; Ramu et al. 2017). A subset of 1,389 individuals were phenotypically characterized across multi-year trials for root hydrogen cyanide content (HCN) as described by Ogbonna and colleagues (2020) using the method previously described by Bradbury et al. (1999).

### Datasets

The initial 3354 germplasm set (annotated GA panel) was subjected to Identity-By-State analysis on genotyping data (see details in “Population structure and genome-wide relatedness” section) and a unique core set of 1536 germplasm was constructed (annotated GU panel). To further guide GA and GU panels germplasm characterization, 62 known South American cassava landraces and wild relatives from the cassava HapMap (Ramu et al. 2017; Bredeson et al. 2016) were included (annotated GUH and GAH panels). To perform spatial population structure analysis, a total of 1657 individuals with georeferenced coordinates were used in addition to the annotated GAHg panel. See Supplementary Table 1 for dataset panels definition.

### DNA extraction

DNA extraction was performed following the protocol described by Albuquerque et al. (Albuquerque et al. 2018). Briefly, DNA was extracted from young leaves according to the CTAB (cetyltrimethylammonium bromide) protocol as described by Doyle and Doyle (1987), with minor modifications. The quantity of DNA was estimated by comparing the fluorescent yield of the samples with a series of lambda (λ) DNA standards (Invitrogen, Carlsbad, CA) at varying known concentrations. The DNA was diluted in TE buffer (10 mM Tris–HCl and 1 mM EDTA) to a final concentration of 60 ng/μL, and the quality was checked by digestion of 250 ng of genomic DNA from 10 random samples with the restriction enzyme *Eco*RI (New England Biolabs, Boston, MA) at 65 °C for 2 h and thereafter visualized on agarose gel.

### Genotyping

Genotyping was performed by preparing pooled libraries using Genotyping-By-Sequencing (GBS) (Elshire et al. 2011) with the *Ape*KI restriction enzymes (Rabbi et al. 2014) and sequenced on the Illumina HiSeq 2500 platform producing read length of 150 bp. Reads were aligned to the cassava version 6.1 reference genome (Bredeson et al. 2016). Single-nucleotide polymorphism (SNP) calling was performed using TASSEL GBS pipeline V5 (Glaubitz et al. 2014). The SNP calling step included the following parameters: quality: -mnQS 1, kmer length: 64 and the taxa key file. Raw output data were subjected to filtering including: Individual-based missing data filtering of 0.8 maximum per chromosome, mean depth values (over all included individuals) greater than 5, missing data up to 0.2 per locus and a minor allele frequency (MAF) of 0.01 per locus. Phasing was performed for each chromosome using Beagle 4.1 (Browning and Browning 2009), a window of 5000 markers and an overlap window of 500 markers. Recombination rate and subsequent haplotype length were taken into account using a genetic map as described in Wolfe et al. (2019). Imputation was performed using the genotype likelihood (GL) mode with 10 iteration steps. Imputed markers were subjected to filtering using an allelic correlation $(A{R}^{2}>0.8)$ equal or greater than 0.8. Dosage format was generated using the pseq library (http://atgu.mgh.harvard.edu/plinkseq/start-pseq.shtml).

### Population structure and genome-wide relatedness

Population structure analysis was performed using loci satisfying Hardy–Weinberg Equilibrium (–hwe: 0.01), MAF (–maf: 0.05) filtering and linkage disequilibrium marker pruning using –indep-pairwise 50 5 0.8 (windows, step, *r*^2^). All filtering was performed using PLINK v1.9 (Chang et al. 2015) and vcftools v4.2 (Danecek et al. 2011). These GBS-based SNP datasets (i.e., GUH and GAHg) were intersected with the whole-genome sequencing (WGS)-based HapMap dataset (available at ftp://ftp.cassavabase.org/HapMapII/rawData/241_accessions/) using the GATK CombineVariants and SelectVariants functions (McKenna et al. 2010).

Germplasm duplicate identification was performed using a set of 9686 SNPs filtered on the basis of genome-wide average proportion of alleles shared between individuals using the IBS estimation approach available in PLINK (Purcell et al. 2007). Secondly, a Ward’s minimum variance hierarchical cluster dendrogram was built from the IBS matrix using the APE v.5.3 package (Paradis 2011) in R (R. Core Team 2015) following the calibration principle approach (Noli et al. 2013; Rabbi et al. 2015) and a reference set of 11 known duplicated accessions. The phylogeny tree was constructed from Identity-by-State (IBS) pairwise distances using hierarchical clustering hclust function (“ward.D2” method) in phyclust v0.1–28 R package version 3.6.3 (2020-02-29) and visualized in FigTree v1.4.4 (http://tree.bio.ed.ac.uk/software/figtree/).

Based on the IBS-selected unique core set of 1536 accessions, population stratification analysis was conducted on the GUH panel and 8242 SNPs using Admixture (Alexander et al. 2009) with fivefold cross-validations for values of K 1 through 20 to define the optimal number of clusters and examine patterns of relatedness and sub-ancestry among our population. Non-parametric approaches, including principal component analysis (PCA) and associated discriminant analysis of principal components (DAPC) using Bayesian information criterion (BIC), were used for validation using the Adegenet package v.2.1.2 in R (Jombart et al. 2010; Jombart 2008). To ensure optimal statistical power for evaluating genetic structure, and to obtain the most accurate number of clusters using DAPC, we kept 500 PCs that explained about 90% of the genetic variance in our dataset, following observations made by Jombart and colleagues (Jombart et al. 2010).

In order to assess family structure and genetic relatedness, identity-by-descent (IBD) estimation was carried out using PLINK as previously described by (Bredeson et al. 2016) for cassava. Briefly, estimation of IBD between two diploid organisms can be highlighted by three subclasses (IBD0, IBD1, IBD2) with probabilities summarized by Cotterman coefficients ($Z0, Z1, Z2$, respectively). IBD0, IBD1 and IBD2 indicate the sharing of zero haplotypes (all four haplotypes distinct), one haplotype (two haplotypes unshared) and two haplotypes (all four haplotypes shared) over a defined genomic region for the two individuals.

### Genetic diversity analysis

Average polymorphism information content (PIC) for the identified genetic groups was computed on 27,045 SNPs using R (R Core Team 2015), according to the methods earlier described by Botstein (Botstein et al. 1980), using the following equation: $PIC = 1-{{\Sigma }_{i=1}}^{n}{{P}_{i}}^{2}-{{\Sigma }_{i=1}}^{n-1}{{\Sigma }_{j=i+1}}^{n}2{{P}_{i}}^{2}{{P}^{2}}_{j}$, where ${P}_{i}$ and ${P}_{j}$ are the frequencies of and $ith$ and $jth$ alleles and $n$ is the number of different alleles for any selected SNP. Observed heterozygosity was estimated using radiator software version 1.1.4 R package (Gosselin T. 2019). MAF were computed using PLINK v2.0 (Purcell et al. 2007), while the fixation index ${(F}_{ST})$ was estimated using Weir and Cockerham’s estimator (Weir and Cockerham 1984). Effective population size $\widehat{{N}_{e}}$ was estimated at an allele frequency of 0.02 using LDNe method (Waples and Do 2008), as described by (Sovic et al. 2019) and implemented in the software NeEstimate v2.0 (Do et al. 2014). To prevent group-size bias on parameter estimates, all population genetic parameters were computed on 120 individuals randomly sampled from each identified group (Supplementary Table 2**)**. The ten genetic groups were identified using DAPC and validated using Admixture as indicated in the population structure section above. Genetic groups (3 and 4) with sample size *n* < 100 were not considered in subsequent downstream analysis.

### Linkage disequilibrium and introgression analyses

Pairwise LD using all markers (27,045 SNPs) with a MAF (frequency of the occurrence of the second most common allele in a population) of 5% on each chromosome was computed using PLINK. In addition, genome-wide LD decay was computed for each identified genetic group. To limit individual group-size incidence on LD estimates, 120 individuals were randomly sampled from each group, identified as a result of Admixture, PCA and DAPC analysis. Population genetics parameters were estimated on aforementioned groups and LD decay curves were fitted using nonlinear regression as previously described (Inghelandt et al. 2011; Bauchet et al. 2017). We estimated the number of SNP markers required to detect large effect association according to the method described by Bauchet et al. (2017).

To identify potential interspecific hybridization between cultivated (*M. esculenta* ssp. *esculenta*) and wild germplasm (*M. esculenta* ssp. *flabellifolia, M. glaziovii*), introgression detection analysis fo*r M. esculenta* ssp. *flabellifolia* and *M. glaziovii* was carried out using the method earlier described by Bredeson and colleagues (Bredeson et al. 2016; Wolfe et al. 2019). Here, we used the whole-genome sequencing HapMap dataset (Ramu et al. 2017) and contrasted groups (cultivated and progenitors) of seven representative accessions each based on the phylogenetic analysis from Ramu et al. (2017), see Supplementary Table 3.

### Spatial population structure, population splits and genome scan for selection

To further investigate the relationship between genetic clusters and available geospatial coordinates, we computed ancestry estimates for the GAHg panel (1657 individuals) and a genome-wide scan using TESS3 (Caye et al. 2016). Briefly, TESS3 is a model-free approach that performs spatial ancestry estimation using least‐squares optimization and on geographically constrained non‐negative matrix factorization. Ancestral genotype frequency matrix G and ancestral coefficient matrix *Q* are used to derive the allele frequencies in the *K* ancestral populations. Locus-specific ${F}_{ST}$ statistics are computed based on the estimated ancestral allele frequencies. The ${F}_{ST}$ statistics are transformed into squared *z*-scores and *p*-values are computed using a Chi-squared distribution with *K* − 1 degrees of freedom (Weir 1997), where K is the number of ancestral populations (Caye et al. 2016). A set of 1039 common accessions between GAHg and GUH panels were used (Supplementary Table 1) to compare results between non-spatial (DAPC) and explicitly spatial (TESS3) population structure analysis. Spatial analysis was also conducted on a set of 702 germplasms that excluded contemporary breeding lines and was collected earlier than year 2000.

To infer possible intermediate stages of domestication, including population splits and migration events, we used 27,045 SNPs on 419 germplasms (Supplementary Table 4). These 419 germplasms are a subset of 702 germplasms in our population collected before the year 2000, excluding contemporary breeding lines, located within different ecoregions, excluding those at the boundary of two ecoregions. We constructed a consensus population graph tree using a SNP window size of 50, five migration events with sample correction disabled, and 1000 bootstrap replications to improve tree robustness. The TreeMix maximum likelihood method (Pickrell et al. 2012), as implemented in the BITE R package version 1.2.0008 (Milanesi et al. 2017), was used to assess graph robustness. The approach is based on population genomics using genome-wide allele frequency and Gaussian approximation to genetic drift.

## Results

### Genotyping-by-sequencing and marker distribution

From the SNP calling, a total of 343,707 initial variant loci were identified. Of these, 30,279 were retained for phasing and imputation after filtering for quality, missing data and MAF. After imputation, 27,045 SNPs with an allelic correlation of $A{R}^{2}$≥ 0.8 were kept for downstream analysis. The marker density of this filtered set on average, one SNP every 19 kilobase pairs (kbp), with a maximum distance of 2 megabase pairs (Mbp). Marker density per chromosome ranged from one SNP every 14 kbp (chromosome 1) to one SNP every 27 kbp (chromosome 7), with an average of 1503 markers per chromosome (Fig. 1b). For IBS analysis on the GA panel, 9686 SNPs remained after Hardy–Weinberg filtering, while 8242 SNPs remained after additional MAF filtering on the IBS-selected unique set (GU).

### Duplicate accession identification and population structure

**Fig. 2:**
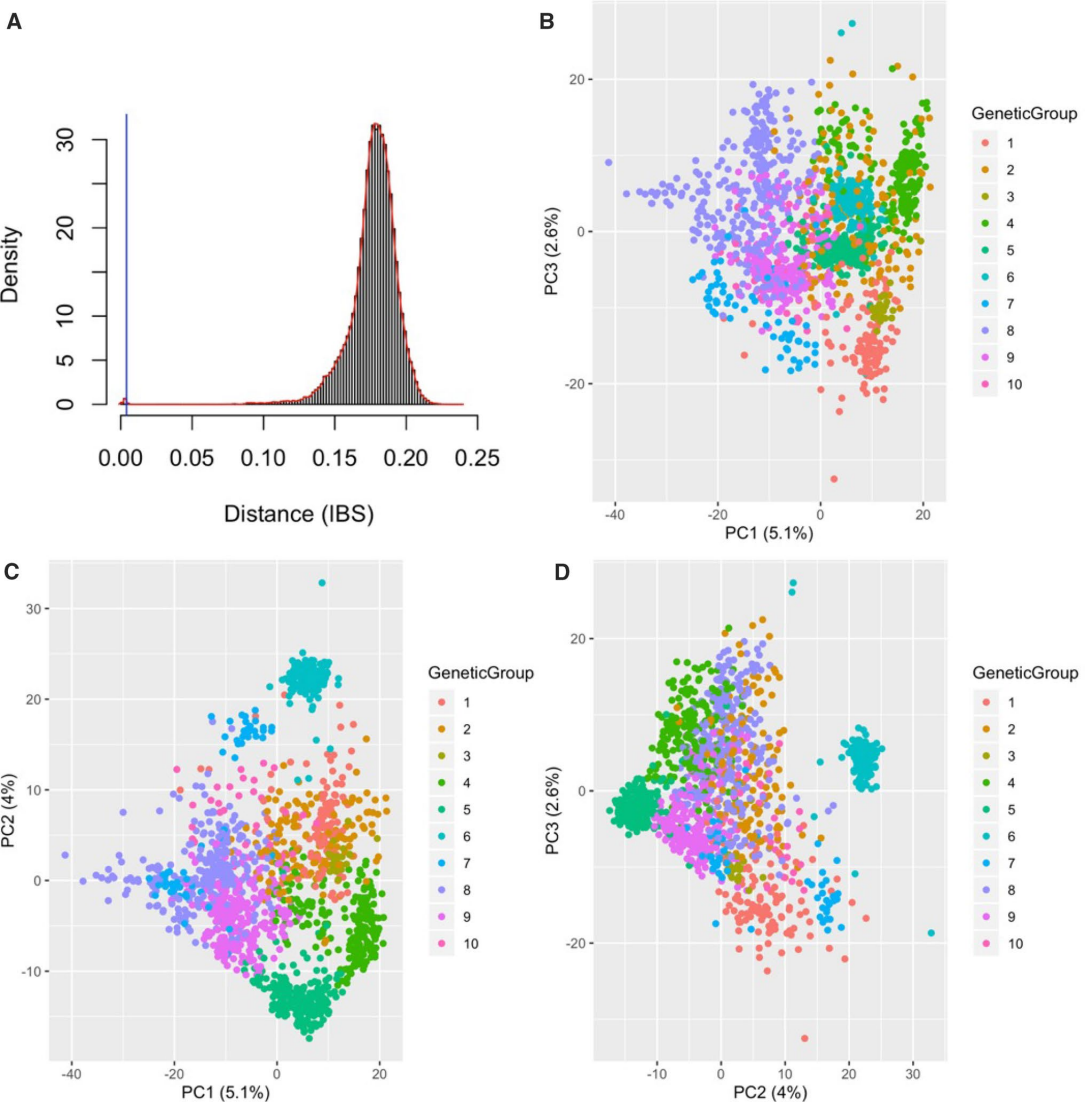
Identity-by-state analysis and population structure. **a** Density plot of IBS values: the left tail shows values close to 0. In IBS scaling, 0 equals identical accessions. The average IBS value for these duplicate accessions was **0.003135969** after excluding the artifacts. Based on a calibration set, we considered the conservative threshold of 0.004, yielding 1818 entries involved in duplication. The blue vertical line denoting the distance threshold below which two samples can be considered identical. **b–d** Principal component analysis of GU (IBS-selected unique set) dataset. Population structure of Brazilian germplasm reveals the first three axes of the principal component analysis (PCA) that explain about 11.1% of the variations in the population of 1536 individuals (GU panel), which includes 8242 SNPs after filtering for Hardy–Weinberg equilibrium and MAF of 0.01 and 0.05, respectively. **(b)** Plot of PC1 and PC3 explaining 7.7% of the variations. **(c)** Plot of PC1 and PC2 explaining 9.1% of the variations. **(d)** Plot of PC2 and PC3 explaining 6.6% of the variations. The color codes represent the genetic groups identified using the DAPC of GUH panel (IBS-selected unique set plus 62 HapMap accessions from Brazilian germplasm)

Because biased representation of duplicated individuals in a dataset will lead to downstream biases in the estimated population genetics parameters, we conducted an identity-by-state (IBS)-based duplicate analysis. This analysis [with Ward's distance threshold of 0.004, based on a calibration set of eleven known duplicated individuals] (Fig. 2a, Supplementary Table 5, Supplementary Figure 1) yielded 1818 duplicates and 1536 unique clusters (annotated as GU panel). Based on available metadata, identified duplicates were distributed across the five ecoregions, with 85% coming from the Northeastern region, covering Cerrado, Caatinga and Atlantic Forest ecoregions, while 13% and 2% came from the Amazonian and Pampa ecoregions, respectively.

**Fig. 3:**
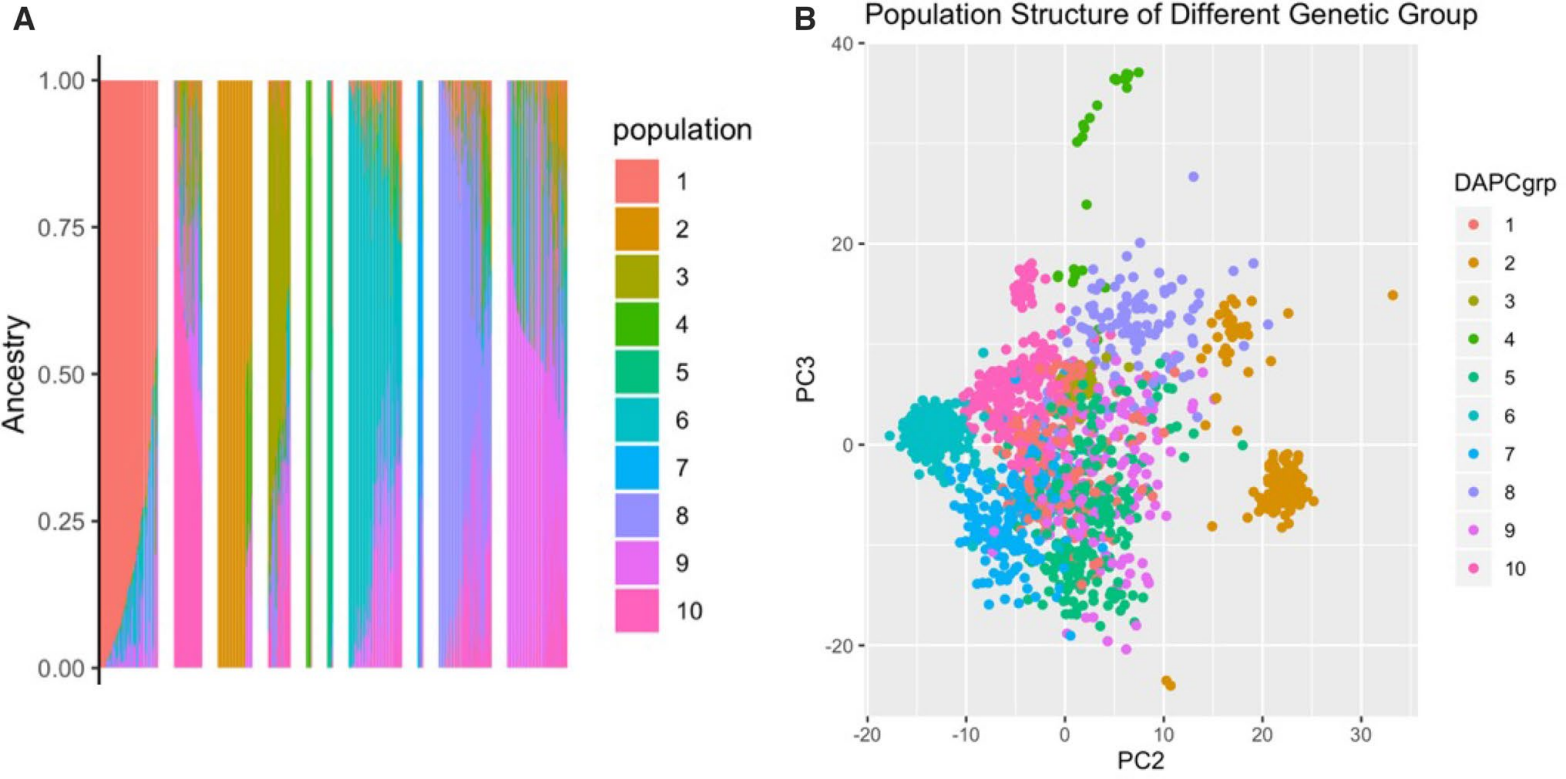
Admixture analysis and discriminant analysis of principal component for GUH dataset with 1536 unique sets and 62 HapMap individuals. **a** ADMIXTURE analysis (*K* = 10) with the probability of assignment ordered sequentially. Proportion of ancestry was plotted on the *Y*-axis against the individual samples on the *X*-axis. **b** GUH dataset PC2 and PC3 view of population structure with individuals colored by their DAPC genetic groups

To further explore the dataset genetic structure, PCA, DAPC and Admixture analyses were conducted. PCA revealed patterns of clustering in GU panel population, with the first three PCs accounting for 11.7% of the genetic variation (Fig. 2b–d). Application of the Bayesian Information Criterion (BIC) identified an optimal number of 10 genetic clusters in the GUH panel (Fig. 3b, Supplementary Figure 2, 3a–b), with group size ranging from 25 to 265 individuals (Table 1). The probability of individual group assignment was 99%, showing stability, indicating that 10 clusters adequately summarize our dataset. The DAPC group identification was further validated using Admixture, a parametric approach with fivefold cross-validation, also identifying 10 subpopulations (Fig. 3a, Supplementary Figure 3c–e). Further analyses and estimation of population genetics parameters were performed on the genetic groups identified in the GUH panel.

**Table 1:** Genetic diversity of Brazilian germplasm

Genetic pools	Group name	Accession # per group	Accessions #sampled	Genetic parameters estimate per group					
				PIC	MAF	F_ST_	Ho	Ne	HCN
Group 1	Admix-Group	155	120	0.154	0.133	0.097	0.29	55.3	−1.08
Group 2	Bahia-Group	166	120	0.133	0.124	0.140	0.39	31.6	0.44
Group 3	Sugary-Group	25							−2.10
Group 4	Wild-type	27							
Group 5	WAXY-Group	255	120	0.159	0.136	0.072	0.31	35.2	−0.45
Group 6	NE-LandRaces	265	120	0.160	0.137	0.106	0.26	53.3	0.81
Group 7	BITTER-Group	194	120	0.163	0.139	0.095	0.29	87.2	1.08
Group 8	SWEET-Group	121	120	0.166	0.143	0.091	0.32	43.8	−0.39
Group 9	NE-Admix-Group	138	120	0.166	0.141	0.074	0.32	10.0	0.27
Group 10	Amazonas-Group	252	120	0.190	0.144	0.078	0.26	31.8	−0.63

The Brazilian germplasm population contains 3345 accessions. Groups 1–10 are based on Discriminant Analysis of Principal Components (DAPC) using the GUH panel. The defined group names are Admix-Group (admixed of improved/landraces from Amazon, Parana, Manaus and Bahia states), Bahia-Group (improved lines from Bahia), Sugary-Group (Sugary cassava Landraces from Northeast), Glaz-Group (wild relatives or *M. glaziovii* and Tree cassava), WAXY-Group (waxy cassava from Amazon), NE-LandRaces (North–North East landraces), BITTER-Group (North East bitter cassava), SWEET-Group (sweet cassava from North East), NE-Admix-Group (admixed of improved/landraces from North East) and Amazonas-Group (landraces from Amazonas). Group 3 and 4 do not have sufficient individuals to be included in further analysis. *Ne* is the estimate of effective population size at allele frequency of 0.02. The model is based on the LD method assuming random mating. The character “#” means number, *PIC* is the polymorphism information content, *H* is the average heterozygosity, and HCN is the average hydrogen cyanide content (based on Best Linear Unbiased Prediction)

To further understand the observed population structure, all individuals were mapped to their available metadata, providing insights into the observed clustering pattern (see Supplementary Table 6). Clustering approaches (Admixture, PCA, DAPC) and metadata on the GUH dataset supported their group identification (See Table 1). Groups 1, 2, 5 and 9 are mixed populations, while other groups (3, 4, 6, 7, 8, 10) have more distinct characterization. Briefly, the 10 groups were annotated (Table 1) as Admix-Group, Bahia-Group, Sugary-Group, Glaz-Group, WAXY-Group, NE-LandRaces, Bitter-Group, SWEET-Group, NE-Admix-Group and Amazonas-Group, respectively (See as well detailed description in Supplementary Note 1). Average root cyanide content per group is shown in Supplementary Figure 3f, with Sugary-Group and Bitter-Group having the lowest and highest of cyanide, respectively (Ogbonna et al. 2020).

### Genetic diversity parameters

**Fig. 4:**
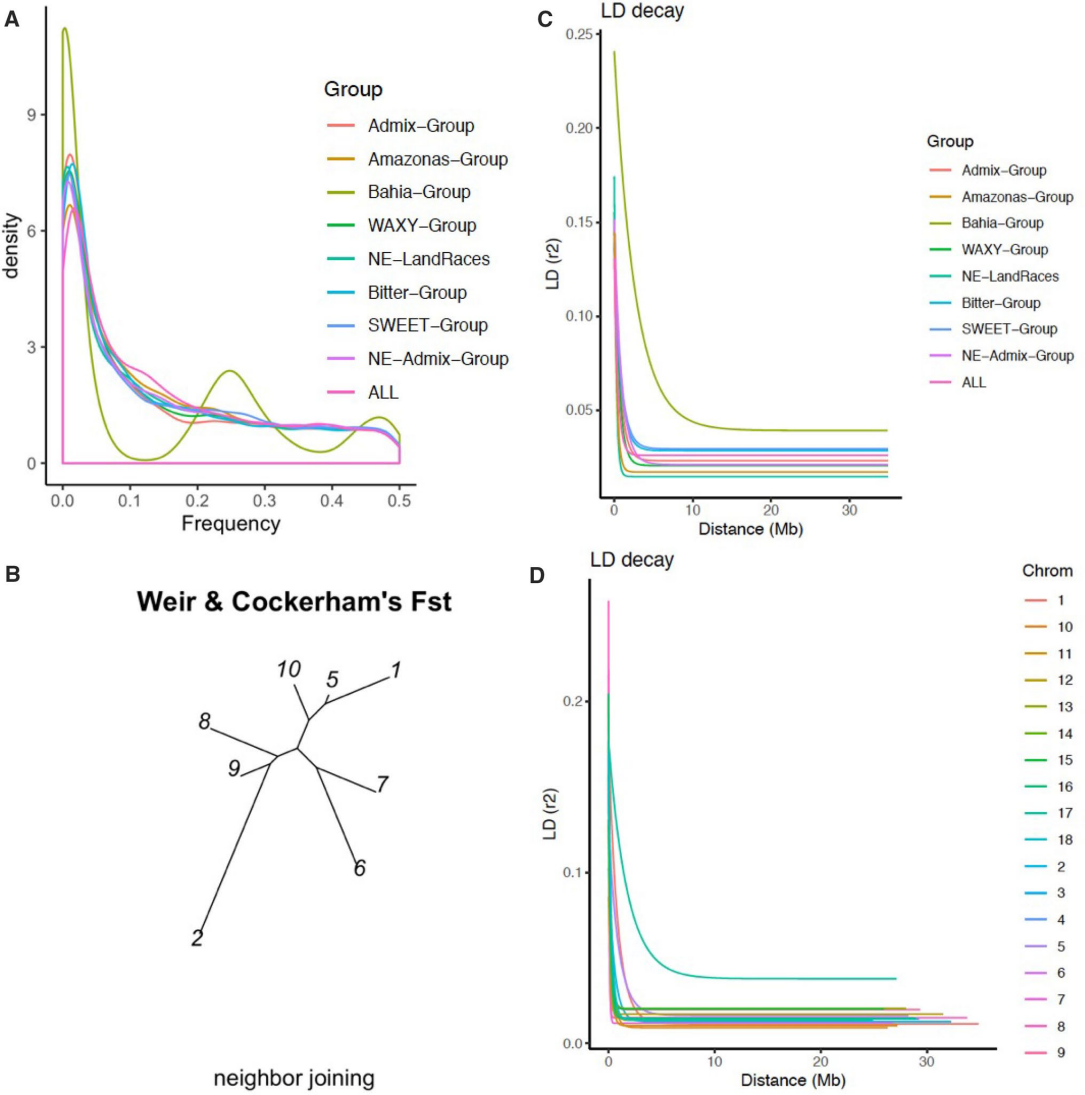
Genetic parameters and linkage disequilibrium on GUH panel. **a** MAF overview across identified genetic groups and combined groups denoted as “ALL”. **b** Neighbor-joining tree showing pairwise Fst of the identified groups in Brazilian population. The groups are 1, 2 and 5 through 10 (respectively: Admix-Group, Bahia-Group, WAXY-Group, NE-LandRaces, Bitter-Group, SWEET-Group, NE-Admix-Group and Amazonas-Group). **c** Linkage disequilibrium decay based on Hill and Weir model (*r*^*2*^) for each identified genetic group and all groups combined accessions, plotted along physical distances across the genome and along the genetic distances in megabase (Mb). Across the whole population is denoted as “ALL” in the legend. **d** Trend line of the nonlinear regression of the linkage disequilibrium measure r^2^ versus physical distance (Mb) between single-nucleotide polymorphism (SNP) marker pairs across chromosomes in the genome of the Brazilian germplasm. The LD is computed chromosome-wide. The average LD *r*^2^ is 0.031 and drops to background level (*r*^2^ < 0.1) at around 300 kb across the genome

MAF across groups showed an average of 0.14, Amazonas-Group and Admix-Group having the highest (0.144) and lowest (0.133) MAF, respectively (Fig. 4a, Table 1). Group pairwise ${F}_{st}$ (Fig. 4b) ranged from 0.037 (Admix-Group and WAXY-Group) to 0.122 (Admix-Group and Bitter-Group), with NE-LandRaces (0.105) having the highest differentiation when compared to the rest of the populations. The overall ${F}_{st}$ was 0.079. Bahia-Group, the family-structured group, was excluded from the reported genetic diversity parameters.

The average PIC across groups was 0.23 (max = 0.375; min = 0.142; Table 1). Approximately, 25% of the SNPs had PIC values greater than 0.3, while 42% had PIC values greater than 0.2 for the population of 3354 accessions. PIC values for identified groups ranged from 0.190 to 0.154, with an average of 0.165. Admix-Group (0.154) showed the least genetic diversity and Amazonas-Group the highest PIC value (0.190) compared to the rest of the groups and also higher than the PIC value of the combined genetic pool. The observed heterozygosity $({H}_{o})$ across groups ranges from 0.26 (Amazonas-Group) to 0.32 (SWEET-Group and NE-Admix-Group) with an average of 0.29 (Table 1). For estimates of effective population size, $\widehat{{N}_{e}}$ ranges from 10.0 (NE-Admix-Group) to 87.2 (Bitter-Group), as seen in Table 1.

### Linkage disequilibrium and introgression analysis

Average genome-wide LD was 0.021 (*r*^*2*^) falling to background levels (*r*^*2*^ < 0.1) at approximately 107 kb (Fig. 3c; Supplementary Figure 4, Supplementary Figure 5, Supplementary Figure 6). All identified groups and chromosomes exhibited average LD (*r*^*2*^) less than 0.1 at genome-wide scale (Supplementary Table 7). Higher chromosomal LD extent was observed on chromosomes 5 and 17 with *r*^*2*^ values of 0.026 and 0.06, respectively, together with the slowest decay (Fig. 4d). Average LD across the genome, excluding chromosome 17, is 0.019 (*r*^*2*^). See Supplementary Note 2 for additional LD details.

The estimated number of SNP markers required to detect QTL with large effect size in association studies showed variation across chromosomes and genetic groups, from 1296 (NE-Admix-Group) to 4713 SNP (genome-wide) markers with *r*^*2*^ = 0.1 threshold 0.1 and from 236 (NE-Admix-Group) to 603 (NE-LandRaces) SNP markers, based on group average LD threshold (Supplementary Table 7).

**Fig. 5:**
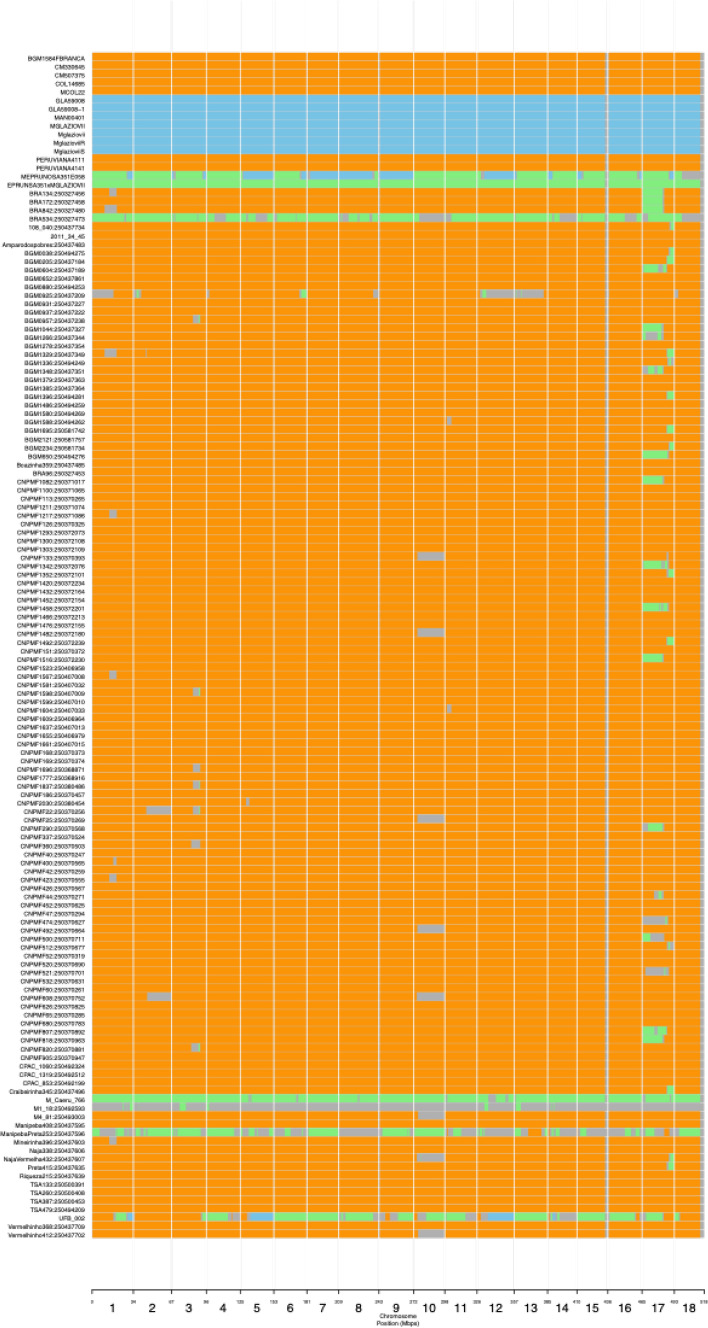
Inferred segmental ancestry of genetic Bitter-Group from Brazilian germplasm collection using Genotyping-By-Sequencing. Bitter-Group is composed of bitter cassava landraces from the Northeastern region of Brazil. Orange color shows both diploid haplotypes are *M. esculenta*; blue is both haplotypes *M. glaziovii*; green, heterozygote haplotypes from *M. esculenta and M. glaziovii*; gray color represents unassigned segments

Introgression analysis using cassava HapMap reference lines for cultivated *M. esculenta* subsp. *esculenta* compared to both wild *M. esculenta* subsp. *flabellifolia* and *M. glaziovii* samples identified 294 (Supplementary Table 8) and 1,795,898 biallelic, ancestry-informative SNP markers (Supplementary Table 9), respectively. These SNP markers are distributed across chromosomes and represent fixed, or nearly fixed, differences between cultivated and wild *Manihot* (Supplementary Figure 8a–b). Of the *M. glaziovii*-specific SNPs, 3238 were represented within the GBS marker set (Supplementary Figure 8c; Supplementary Table 10), which were used to infer ancestral segments in the identified genetic groups. Introgression of *M. glaziovii* in the Brazilian germplasm collections was found on two chromosomes. The largest introgressed segment (16.8 Mb size, in interval 458,800–17,307,782 bp) was found in chromosome 17, and predominately in the Bitter-Group (Fig. 5). The Bahia-Group and the SWEET-Group contained smaller introgression segments (1.1 Mb size, in interval 5,755,106–6,884,548 bp) on chromosome 5 (Supplementary Figure 9).

### Spatial population structure and genome scan for selection

**Fig. 6:**
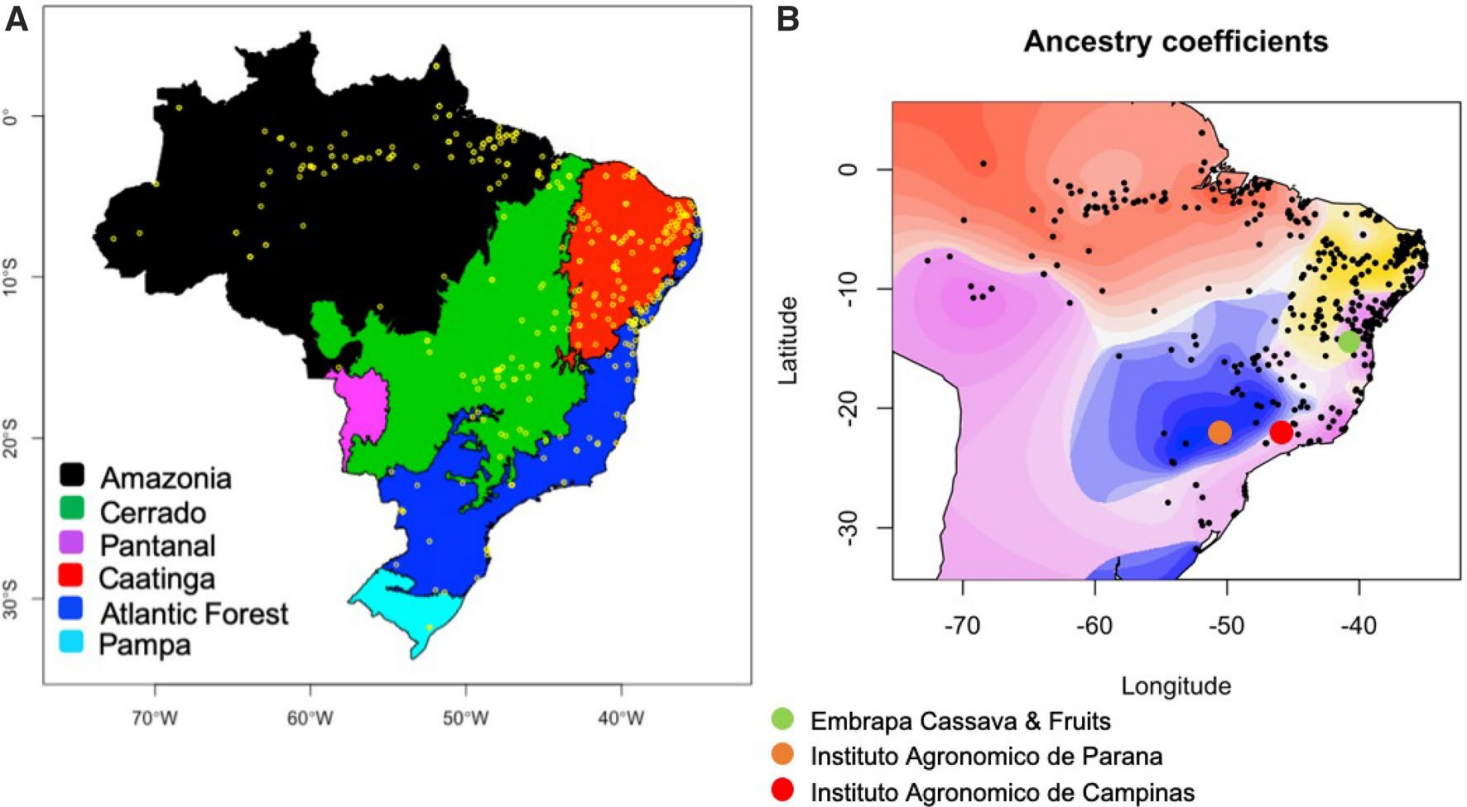
Brazilian ecoregions and spatial analysis of GAHg panel (1657 accessions) using tess3r. **a** Map of Brazilian ecoregions highlighting the different biomes, including yellow dots representing the germplasm collection-point coordinates. **b** Geographic maps of ancestry coefficients for *K* = 5 ancestral populations. The black dots represent the germplasm collection points in Brazil. The fifth cluster is not strongly localized, hence hidden. Colored dots (red, orange, and green) represent the geographical locations of the main Brazilian breeding programs active in the past 40 years

The number of ancestral populations, $K$, was chosen after evaluating the cross-entropy criterion curve (Frichot et al. 2014), which exhibited a monotonic decrease and plateaued at $K$ = 9 (Supplementary Figure 10a). Spatial analysis detected five major ancestral populations and nine extent populations (Supplementary Figure 10a). The nine clusters detected in this spatial analysis in comparison with the ten clusters detected using DAPC and Admixture were as a result of missing individuals in the Glaz-Group, given that they had no georeferenced coordinates. We assessed the interpolation of ancestral populations of *K* = 4 through *K* = 9. Between $K$= 4 and $K$= 5, an elbow in the curve was observed ($K$= 5, green line). For $K$ greater than 9, cross-validation scores decayed until $K$= 20. Corresponding ancestry coefficients estimate (shown in a biplot; Supplementary Figure 10b), interpolation on a geographic map of Brazil was produced for $K$= 4 to 9 ancestral groups (Supplementary Figure 10c). Evaluating the interpolated ancestry at $K$= 4, grouped individuals from Caatinga and Cerrado ecoregions grouped together. At *K* = 5 through 7, five stable groups were resolved; the individuals from Caatinga and Cerrado differentiated into two ecoregions and individuals in the Pampa ecoregion grouped together with Cerrado (Supplementary Figure 10c, Fig. 6a). At *K* = 8 and $K$= 9, there were additional differentiations (two zones) within the Cerrado and the Atlantic Forest ecoregions. (Supplementary Figure 10c, Fig. 6a).

The interpolated ancestry resulting from the five stably-formed groups (from *K* = 5 through 7) presented strong geospatial overlap with the six Brazilian ecoregions (IBGE 2019); excluding the Pantanal ecoregion, where no samples were collected for this study (Supplementary Table 11). In Fig. 6b, the region in yellow/shades is associated with the semi-arid region of the Brazilian Northeast with shallow and stony soil, sparse vegetation and low levels of annual rainfall, while the region in red/shades represents the coastal region with high annual rainfall, deeper soils, with higher and dense vegetation. The fifth cluster in Fig. 6b is not strongly localized, hence was hidden, thereby displaying only four clusters/colors of ancestral population coefficients.

Using 702 landraces (excluding breeding lines), collected before the year 2000, distributed across the five ecoregions of Brazil, the genetic groups show strong overlap with the ecoregions (Chi-squared statistics: X-squared = 593.65, df = 12, *p*-value < 2.2e–16) (Supplementary Figure 11, Supplementary Table 12).

A genome-wide scan for selection, based on population differentiation in the inferred ancestral populations from spatial analysis, detected 24 loci above the Bonferroni-adjusted threshold (Supplementary Figure 12, Supplementary Table 13**)**. The average percentage of accessions grouped in the same groups between GUH panel DAPC and spatial analysis GAHg panel was 85% and ranged from 96 to 53% with only two groups having less than 86% (Bitter-Group: 77% and SWEET-Group: 53%), given that GAHg panel contains 1036 individuals in common with GUH panel (Supplementary Table 14).

**Fig. 7:**
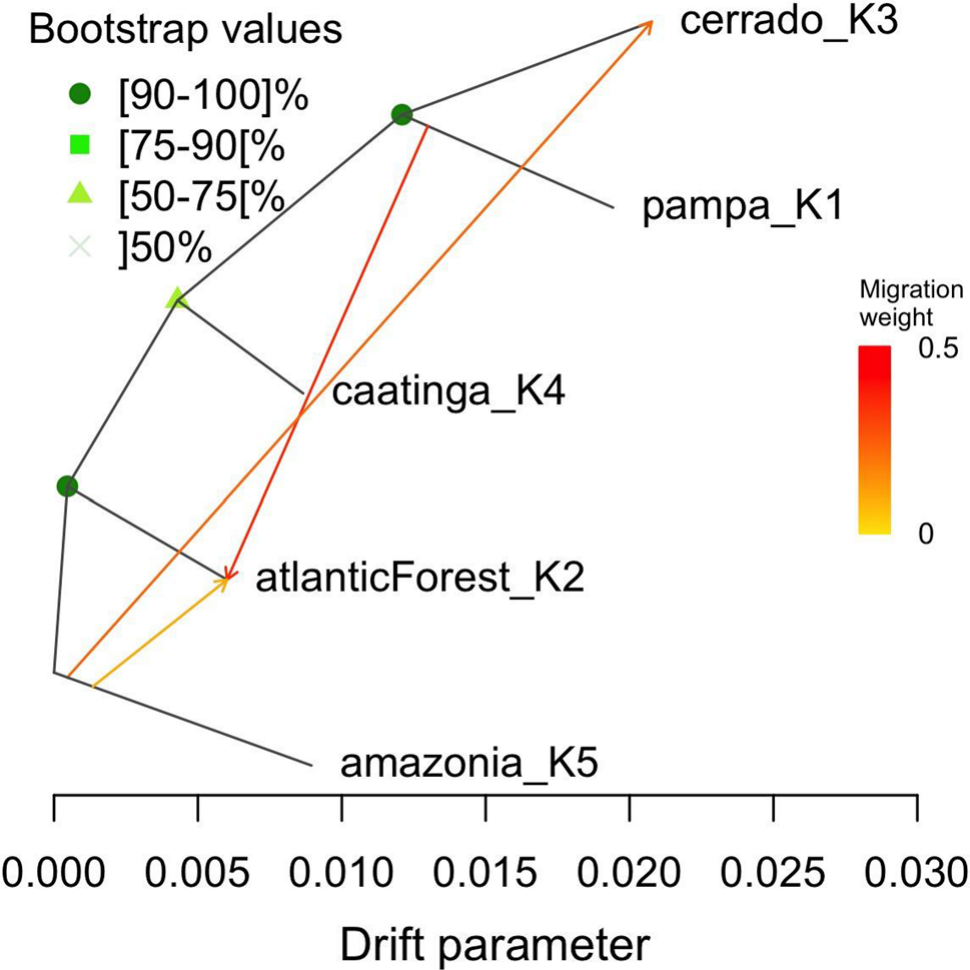
Gene flow analysis using TreeMix (v1.12) for 419 Brazilian germplasm collected before the year 2000. Plotted is the structure of the graph inferred with gene flow events of 3 migration using Maximum likelihood. Individuals in the 5 populations from 5 different Brazilian Ecoregions. Migration arrows are colored according to their weight. Horizontal branch lengths are proportional to the amount of estimated genetic drift. Nodes robustness was estimated with 1000 bootstrap replicates. The analysis was carried out under the implementation of BITE R package version 1.2.0008

TreeMix gene flow analysis using 419 accessions showed three population splits with node robustness values of 1000, 636 and 961, respectively, out of 1000 bootstrap replications. Three migration events were thus identified with 0.999 variance explained, indicating the goodness-of-fit of the model with gene flow from Amazonia to Caatinga, Amazonia to Cerrado and Pampa to Atlantic Forest ecoregions. The Atlantic forest ecoregion had the least genetic drift, followed by Amazonia and Caatinga, and then Pampa and Cerrado ecoregions, respectively (Fig. 7).

## Discussion

Using 27,045 SNP markers obtained from the Genotyping-by-sequencing (GBS) method, we identified and characterized genetic groups of the Embrapa Brazilian cassava germplasm collection and inferred the parameters describing genetic diversity, population structure and linkage disequilibrium.

### IBS Duplicate identification

Accession mislabelling has been reported to reduce the genetic gain of cassava by up to 40% in Africa (Yabe and Jannink 2018). In order to address germplasm duplication in the Brazilian collection, we inferred genetic identity between accessions using an identity-by-state (IBS) analysis. Pervasive identity between accessions was indeed observed among the Embrapa collection, identifying 1818 (54%) duplicates out of the 3354 initial accessions examined in this study. The main outcome of the IBS analysis is that (1) a unique set of 1536 accessions were identified (GU panel) using Ward’s distance threshold of 0.004 (Fig. 2a, Supplementary Table 1); (2) This would reduce biases from over-representation and genetic contribution of duplicated individuals to genetic variance in a population. However, our motivation in this study for duplicate analysis was to inform efficient germplasm and resource management at the Brazilian cassava program and balance individual genetic contribution to population structure definition. About 85% of the duplicated individuals were found within the Northeastern region, this region hosts the main cassava breeding programs in Brazil, highlighting the impact of germplasm exchange and adoption of improved varieties by farmers, which are often renamed.

### Population genetic and geospatial structure

The characterization of this unique set of 1536 accessions yielded DAPC structure patterns of 10 genetic groups, corresponding to the BIC profile obtained from a stepping stone model as described by Jombart et al. (2010). Previous studies from Embrapa reported more genetic groups (> 20) with some of the groups having less than ten accessions (Oliveira et al. 2014; Albuquerque et al. 2018). The lower probability of individual group assignments (PAI) from DAPC can be related to highly admixed individuals sharing haplotypes segments from multiple clusters identified (Albuquerque et al. 2018). The aforementioned studies could not however establish identified population structure and geographical origins. The germplasm used in this current study was collected from the 26 states (including Federal District) and has a broader origin representing five ecoregions of Brazil, suggesting the observed population structure may have been influenced by breeding activities, and geographical boundaries and restrictions (Fig. 1a, Fig. 6a).

In the past 40 years, breeding activities have mainly taken place in the Northeast and Southeast regions of Brazil (Fig. 6a, green, red and orange dots) while the Amazonian region maintained a more traditional approach to cultivation and breeding of cassava (Mckey et al 2010). These facts are further supported by the observed germplasm and genetic diversity distribution patterns across Brazil, in which the Coastal and Cerrado regions have been more accessible to prospect than the Amazonian region (Fig. 6b, Supplementary Figure 3b-e). This observation was further supported by the ancestral coefficient estimated via Admixture, where germplasm within the Amazonian region had less diverse ancestral coefficient than germplasm within the Central-West and Southeastern regions of Brazil (Cerrado and Atlantic Forest ecoregions) (Supplementary Figure 3d–e). Based on these elements, we speculate that a more diverse population has been collected in Cerrado, Atlantic Forest and Caatinga than in the Amazon.

Non-parametric methods such as PCA and DAPC can provide accurate population structure estimates in complex scenarios (i.e., continuous admixture, complex substructure) and lead to improved population inference. Admixture models, however, are more interpretable when using spatial information (François and Durand 2010). When combined, these approaches provide a platform for understanding the spatial distribution of adaptive and nonadaptive genetic variation in different organisms (Manel et al. 2010). Spatial analysis admixture coefficients from tess3r analysis provided clear evidence for the clustering of homogeneous genetic groups in Brazil ecotypes/ecoregions. The interpolation and projection of ancestry coefficient to the geographic map of Brazil suggested a clinal variation along different biotypes/ecoregions across Brazil with a distinct ecotone between ecoregions. Cross-validation of co-ancestry coefficients underlined a strong decay between $K=4$ and $K=5$, suggesting a significant past event such as domestication, environmental selection or modern breeding activities leading to group differentiation. Interestingly, individuals initially grouped together at $K=4$ between the Northeast (Caatinga) and Central-West (Cerrado) ecoregions (Supplementary Figure 10a), split into ecoregion-specific groups. This hypothesis is further supported by the observed divergence region located within the regions of breeding activities in the last 4 decades.

Recently, Muhlen et al. (2019) hypothesized two groups each of sweet and bitter cassava using a country-wide sample of 494 cassava landraces covering 11 geographic regions of Brazil. In this current study, spatial analysis revealed five genetic groups were identified with substructures observed from $K=6$ through 9. These substructures mostly appeared within the Cerrado, Caatinga, Atlantic Forest and Pampa ecoregions, highlighting regions of active cassava breeding in Brazil. The nine genetic groups identified using tess3r were as a result of having only 1039 individuals from the GUH panel with georeferenced coordinates. Georeferenced individuals clustered in 9 groups and matched the groupings observed in the DAPC GUH panel, except for Glaz-Group (group 4) where individuals are wild relatives from whole-genome sequencing HapMap accessions (Ramu et al. 2017). The average percentage of matching group accessions was 85% across groups and indicates similarity in group assignment between the two independent structure analyses (Supplementary Table 14). Therefore, spatial analysis results were in large part congruent with our initial Admixture and DAPC 10 clusters partitioning of the GUH panel Supplementary Table 14). Observed patterns were broadly consistent with an isolation by distance scenario, in which genetic similarity decayed with geographic distance (Supplementary Figure 3a, c), as described in the DAPC stepping stone model (Jombart et al 2010).

Mühlen et al. (2019) also reported that sweet cassava landraces were dispersed southward through the Cerrado where they formed a genetically distinct group, different from sweet cassava landraces in Amazonia and coastal Brazil. From our spatial analysis, especially at *K* = 5, we speculate that the significant divergence observed between the Caatinga (Northeast region) and Cerrado—Pampa (Central-West and South regions) ecoregions may represent the second phase of cassava domestication from initially “sweet” domesticated cassava to “sweet and bitter” cassava (Fig. 6b). This hypothesis was previously suggested by Arroyo-Kalin (2010) and further supported by Mühlen and colleagues (2019); however, the present study provides the first novel evidence of cassava domestication using spatial analysis and further evidence of divergence of an ancestral population into two ancestral groups.

Spatial distribution of genetic groups of cassava in Brazil is different across bitter and sweet cassava, owing to haplotype diversity and environmental heterogeneity of cassava-growing regions (Alves-Pereira et al. 2018). This is captured particularly in the average cyanide distribution across identified genetic groups (Supplementary Figure 3f). Mühlen and colleagues (2019) reported the diverse distributions of sweet and bitter cassava across Brazil, based on germplasm mostly collected before year 2000 and concluded that it does not coincide with the Brazilian ecogeographic regions. To test this hypothesis, we performed spatial analysis strictly using 702 landraces accessions, with the collection year prior to year 2000. The distributions of ancestral coefficients shown in Supplementary Figure 11 exhibited a genetic structure overlapping Brazilian ecoregions boundaries, while highlighting how recent migration/germplasm exchanges modified the genetic landscape of cassava across Brazil.

### Genetic diversity estimation

We further characterized the identified genetic groups based on inferred parameters describing genetic diversity. Firstly, MAF distributions across identified groups exhibited variation skewed toward intermediate frequencies (MAF 5–20%) in some groups (SWEET-Group, Amazonas-Group) and a higher amount of rare (< 5%) alleles in others (Admix-Group, Bahia-Group, NE-LandRaces, Bitter-Group), suggesting semi-isolated groups. The Amazonas-Group showed the highest diversity (PIC = 0.190) and rare alleles richness (MAF = 0.144) captured in our dataset, consistent with the origin of cassava in Amazonian ecoregion (Allem 2001; Clement et al. 2010). Genetic diversity showed a decrease from the Amazonas via NE-Admix-Group (PIC = 0.166), to groups such as the SWEET-Group, BITTER-Group and NE-LandRaces (PIC = 0.166, 0.163 and 0.160), in Northern and Northeastern region; diversity further decreased in the WAXY-Group (PIC = 0.159), in Cerrado ecoregion, to Admix-Group (PIC = 0.154), consisting of individuals from across Brazil. Decreasing numbers of minor alleles were observed along this gradient, suggesting successive small founder populations (i.e., a stepping stone model).

Overall, the MAF identified in the germplasm included in this study provides sufficient power for association studies in a panel that includes a broad range of genetic diversity. The average MAF of 0.14 (14%) indicates that most loci (SNPs) in this study have required allele frequency (> 0.05) for association (and other related trait mapping) studies. MAF highlights informative markers, rare variants and alleles conservation in a population. Our result falls within the range reported for other clonal crops like banana (Sardos et al. 2016) and potato (Sharma et al. 2018).

Additionally, Pairwise ${F}_{ST}$ (0.073) reflects a lower population differentiation and a moderate impact of domestication of the crop compared to other South American domesticated crops such as tomato (Razifard et al. 2020). The highest level of genetic differentiation (0.108) was observed between the SWEET-Group and NE-LandRaces, implying that breeding activities may have restricted gene flow between them and the rest of the groups (Fig. 4b). Average ${F}_{ST}$ with a value range of 0.073 (excluding bottlenecked Bahia-Group) and 0.118 (including bottlenecked Bahia-Group) showed a similar variation to maize (Yu et al. 2006). In cassava, an average ${F}_{ST}$ of 0.040 based on EST-derived SNP markers was reported for cassava germplasm and breeding lines collections of CIAT and African populations (Ferguson et al. 2019), although lower than observed within Brazilian populations. Ferguson and colleagues reported an average ${F}_{ST}$ of 0.013 between South American CIAT breeding lines and germplasm collections, indicating a slight differentiation (Ferguson et al. 2019). However, our study identified within Brazil, across identified genetic groups, ${F}_{ST}$ range of 0.037 and 0.122, indicating higher differentiation (more diverse groups) when compared to CIAT, Colombia.

In addition, polymorphism information content $(PIC)$ indicates measures of genetic diversity of a genomic segment which elucidates evolutionary pressure and mutation rate in a population over a given period of time (Botstein et al. 1980; Niu et al. 2019). $PIC$ values for this study (ranges from 0.142 to 0.375 and average of 0.18) are in congruence with the maximum $PIC$ value of 0.375 for biallelic loci (Nei 1987; Botstein et al. 1980; Luo et al. 2019). Previous studies in cassava reported average $PIC$ values of 0.26 (Oliveira et al. 2014), 0.24 (Albuquerque et al. 2018) using SNP markers. In this current study, we only used biallelic SNP, hence the lower $PIC$ value estimated when compared to previous studies in Brazil. The lower average $PIC$ value could also be attributed to low mutation rate in our population (Coates et al. 2009; Eltaher et al. 2018). $PIC$ of 0.177 indicates moderate genetic diversity in our population, given an expected maximum value of 0.375 for markers that are biallelic. Furthermore, observed heterozygosity was higher for genetic groups having lower effective population size (Table 1) and this indicates the capability of those identified genetic groups to immediately respond to selection; however, the amount of allelic variation left is important for long-term response to selection and survival of those populations (Allendorf 1986; Sovic et al. 2019). In addition, allelic diversity is generally more sensitive to drift than heterozygosity (Allendorf 1986; Sovic et al. 2019). This is made possible in a clonally propagated crop like cassava by incorporated seedlings into clonal stocks to provide new genotypes and additional allelic variation (McKey et al. 2010). We generally observed high heterozygosity across identified genetic groups, indicating a high proportion of genetic variance (allelic variation) across locus in our populations. Taken together, these results show that Admix-Group, Bitter-Group, Amazonas-Group and NE-LandRaces exhibited a higher genetic diversity compared to the rest of the groups and may be explored for heterotic responses in future breeding efforts as recently developed (Peprah et al. 2020).

Finally, an estimate of effective population size, $\widehat{{N}_{e}},$ was estimated using an allelic frequency of 0.02 in order to increase precision and minimize bias (Do et al. 2014). $\widehat{{N}_{e}}$ from the identified genetic groups indicates the ideal population size for which an equivalent level of genetic drift to the real population is lost in Brazilian germplasm and may predict the ability to respond to future changes/breeding strategy employed in the use of these genetic materials in future breeding efforts. These germplasm collections also serve as a gene bank and are very often reintroduced into the breeding pipeline to conserve diversity or breed for a target trait. The estimate of ${N}_{e}$ gives a better understanding of the present and future importance of genetic drift of Embrapa cassava populations and useful information of in situ germplasm management in Brazil. The Bitter-Group shows greater genetic diversity and less drift, followed by Admix-Group and NE_LandRaces, while the rest had $\widehat{{N}_{e}}$ less than 50, making them predisposed to genetic drift. However, it has been shown that small effective population size of a genetic pool indicates high LD and potential for improved prediction accuracy (Yabe et al. 2018a, b). Applying the 50/500 rule as described by Franklin and colleagues (Franklin and Frankham 1998), a genetic group’s effective population size of at least 50 individuals is necessary for conservation of genetic diversity in the short term and avoids inbreeding depression. While a genetic group’s effective population size of at least 500 individuals is required for long-term survival with the ability to evolve under a changing environment and avoiding serious genetic drift. Minimizing loss of genetic variation via genetic drift can be partly achieved by minimizing inbreeding as a result of equalization of family sizes in germplasm collections and regeneration. This would involve implementing breeding strategies that take into account family sizes, co-ancestry and long-term preservation of diversity in developing a population, by emphasizing optimal genetic contributions of parental selections for long-term genetic gains.

Studies with domesticated animals and plants have shown that the loss of genetic variation has negative effects on growth, survival, development and also reduces the evolutionary potential of a population (Allendorf 1986; Greenbaum et al. 2014). This is also important for germplasm collections that serve as a gene bank for possible reintroduction into the breeding program. In this study, we measured genetic variation and estimated effective population size across identified genetic groups to inform optimized germplasm management and conservation of genetic variability for prospecting of breeding approaches in a cassava breeding program. Breeding schemes should be designed to increase effective population size, thereby minimizing genetic drift in cassava germplasm in Brazil. Genetic variability fuels breeding innovation. In an ever-increasing globalization context, public and private breeding initiatives foster germplasm exchange to solve key breeding challenges (i.e., disease resistance). With Brazil as a center of diversity and Africa as the main production zone, the cassava breeding community illustrates well this challenge. Such action implies a better characterization, knowledge of genome landscape (LD) and tracking of cassava germplasm, including reassignment of new names to exchanged germplasm in breeding programs.

### Linkage disequilibrium and introgression in Brazilian germplasm

LD in cross-pollinated species decays more rapidly than among self-pollinated species as a result of more effective recombination that occurs in the former (Zhu et al. 2015). The extent of whole-genome LD in this study ($\sim$ 107 kb; *r*^*2*^ < 0.1) was higher than previously estimated in Brazilian cassava ($\sim$ 20 kb; *r*^*2*^ < 0.2) (Albuquerque et al. 2018), East–West African ($\sim$ 50 kb; *r*^*2*^ < 0.2) datasets (Wolfe et al. 2016) and for HapMap (3 kb; *r*^*2*^ = 0.1) (Ramu et al. 2017) but lower compared to West African ($\sim$ 2 Mb; *r*^*2*^ < 0.1) (Rabbi et al. 2017). Among other outcrossing species, LD observed in this study was higher than LD observed for global panel in maize ($\sim$ 10 kb; *r*^*2*^ < 0.1) (Yan et al. 2009), sweet potato (0.6 kb; *r*^*2*^ < 0.1, 1.2 kb; *r*^*2*^ < 0.2) (Wadl et al. 2018), yam (100 bp; *r*^*2*^ < 0.1) (Akakpo et al. 2017) and potato ($\sim$ 275 bp; *r*^*2*^ < 0.1) (Stich et al. 2013).

The comparison of average LD decay threshold to the standard threshold of 0.1 (*r*^*2*^) for all genetic pools shows a similar trend with average genetic pool’s LD decay having higher genetic distances across groups (Supplementary Figure 5b). Given the allogamous nature of cassava, the pattern of LD may reduce prediction accuracy, since LD is crucial for genomic selection (GS) breeding (Jannink 2010; Yabe et al. 2018a, b). However, the high LD observed in this study indicates potential for improved accuracy using this population for GS breeding.

Genomic variation across the genome and within groups (excluding family-structured group, Bahia-Group) reveals that on average, based on the LD decay (*r*^*2*^ < 0.1), 1300–4700 SNP markers would be needed to detect large QTL association, while the SNP density of 27,045 was used in this study with 19,085 bp average distance between two SNPs, a much higher marker density will be required to detect small effects QTL association (Supplementary Table 7).

Based on the observed genome-wide LD landscape, we speculated that the extended LD observed in chromosome 17, influencing the LD decay for most population groups except Amazonas-Group **(**group 10; Supplementary Figure 6b), was an introgressed segment. Earlier studies reported that Latin American samples from CIAT showed almost no evidence of *M. glaziovii* introgression on both chromosomes 1 and 4 (Wolfe et al. 2019), where introgressions were detected for African materials (Bredeson et al. 2016; Wolfe et al. 2019). Using the HapMap WGS dataset, we identified “pure” *M. esculenta* subsp*. flabellifolia*, *M. esculenta* subsp. *esculenta* and *M. glaziovii* accessions (Ramu et al. 2017), with *M. esculenta* subsp. *esculenta* accessions originating from Brazil. In this study, the identified variants (294) were too few and too sparse to assign segments of *M. esculenta* subsp*. flabellifolia* ancestry across the genome, leading to the speculation that the observation may be incongruent with the evolutionary hypothesis that *M. esculenta* comes from *M. flabellifolia,* indicating that the two species may be too close to identify long, contrasted introgression segments. Following the evolutionary history example of another South American crop, tomato (autogamous), cassava does not present such a strong domestication. Therefore, lower differentiation and subsequent contrast between its wild ancestor and contemporary cultivated lines may limit the prevalence of private alleles between *M. flabellifolia* and *M. esculenta* groups. Identification of structural variants (i.e., indels, duplications) would help in that regard.

On the other hand, comparing *M. glaziovii* to *M. esculenta* Brazilian germplasm, we identified a large set of differentiating markers, in contrast with the few differentiating markers earlier reported for CIAT germplasm (Wolfe et al. 2019). The observed 3238 intersecting SNPs in our GBS dataset may indicate evidence of past or more recent introgression in Brazilian germplasm (Supplementary Figure 8b, c). Bredeson and colleagues (2016) hypothesized that some of the 98 described species of *Manihot* (Miller et al. 1972; Nassar et al. 2008) may represent interspecific hybrids or admixtures (Bredeson et al. 2016). This hypothesis was further developed by Ramu et al. (2017). In the current study, we found individuals that are interspecific hybrids of *M. glaziovii* × *M. esculenta* (“MEPRUNOSA351E058” and “EPRUNSA351xMGLAZIOVII”), and another individual (“UFB_002”) that appears to be from a “pure” *M. esculenta* × *M. glaziovii* hybrid backcrossed to another *M. glaziovii* (or, alternatively, a hybrid that includes *M. esculenta* that already had introgression), supporting the hypothesis put forward by Bredeson et al. (2016). Accession “UFB_002” and a couple of others (“M1_18:250,492,593” and “ManipebaPreta253:250,437,596”) share alleles with neither of *M. esculenta* or *M. glaziovii*, meaning, they may have hybridized with a third species. In addition, Bredeson and colleagues (2016) found an unidentified ancestral segment shared by two Brazilian accessions in chromosome 2 and could not confidently assign their ancestry based on the collection of *M. esculenta* and *M. glaziovii* alleles used in their study. We found evidence for introgression of *M. glaziovii* in Brazilian Germplasm collections on chromosome 17 and particularly in the genetic Bitter-Group (Fig. 5), confirming our initial speculation of introgression on chromosome 17 based on LD landscape. The individuals in Bitter-Group represent landraces of bitter cassava (see Supplementary 3f for average HCN per group) from Northeastern region of Brazil, indicating that the introgression segment we observed may have been as a result of past breeding effort rather than a more recent breeding activity (Allem 2001). Wolfe and colleagues (2019) reportedly found a segment of *M. glaziovii* in three CIAT accessions originating from Brazil within a few clones (“BRA134:250,327,456”, “BRA172:250,327,458” and “BRA842:250,327,480″). We could confirm this finding, with these accessions clustering within the Bitter-Group. Explaining the ancestral segments in Fig. 5**,** with blue and green segments highlighting the presence of *M. glaziovii* introgressions, specifically in individuals identified as hybrid including “Manipeba,” clones showing feral characteristics form of cassava from Northeast Brazil, hypothesized as a transitional link between wild ancestor and cultivated cassava (Allem 2001; Albuquerque 1969). Subsequently the high $\widehat{{N}_{e}}$ observed in the Bitter-Group may be inflated by such introgression. Wolfe et al (2019) observed a homozygote deficit of *M. glaziovii* introgressions in African elite clones and landraces. In the present Brazilian dataset, including both elite material and landraces, *M. glaziovii* inferred introgression segments were only identified in the heterozygote stage. The absence of homozygote *M. glaziovii* introgressions in the studied Brazilian germplasm collection and the fact that *M. glaziovii* African hybridization events were generated by modern breeding efforts (Nichols 1947), let us further speculate: (i) a heterozygous advantage of *M. glaziovii* introgressions in both African and Brazilian germplasm, (ii) the counter selection of homozygote M. glaziovii introgressions under natural/anthropic selection pressure and (iii) *M. glaziovii* hybridization events in Brazilian germplasm might be older than in African germplasm.

### Genome scan for selection

Substantial natural and artificial selection was revealed by the genome scan for selection, highlighting genetic variation of cassava collection in Brazil. The Manhattan plot exhibited islands of strong differentiation around positions in all chromosomes (except chromosomes 3, 6, 8, 10, 11, 14 and 17), with 24 differentiating loci identified across the genome**,** indicating beneficial allele and directional selection in the genome (Wolfgang 2019)**.**

Crop domestication in most cases involves intermediates that are unexplored or underexplored corresponding to historic evolutionary stages in domestication events, and cassava is not an exception. The Southwestern Amazonia indicates a region around the center of domestication of cassava as was earlier hypothesized and supported by (Olsen and Schaal 1999, 2001; Olsen 2004; Mühlen et al. 2019). Estimation of population trees with admixture using Treemix and robustness assessment of the inferred nodes in Fig. 7 as well as the fraction of the explained variance statistically supports the identified population splits and gene flow events. Walker et al. (2019) showed the source of the rivers that feed the Amazonian ecoregion’s rivers and its connections to other ecoregions within Brazil **s**uggesting the likely connections between the Amazonian ecoregion and the Amazon basin, leading to potential routes of seeds/cuttings migration or dispersion by animals or other elements. Given that Northern Bolivia and Amazonian (Clement et al. 2010) shows ancient tracks of cassava domestication, this supports the observed gene flow events, with migration through the Amazonas to the rest of the ecoregions potentially via the river that connects to the Amazon basin. This tree seeks to suggest a second population split between Caatinga and Atlantic forest. However, Caatinga seems to be slightly earlier than Atlantic forest based on the genetic drift parameter, leading us to suggest that cassava may have been propagated to the coastal part of Brazil independently of its migration through the Amazon region. This hypothesis is supported by an independent spatial analysis of this set of individuals, with Southern Amazonas showing the same ancestral coefficient as those in the coastal region of Brazil (Supplementary Figure 11b). The Atlantic forest ecoregion may have experienced less genetic drift given that breeding activities had mostly occurred around that region in the last four decades. The gene flow from Amazonas to Cerrado and Atlantic forest is consistent with Olsen and Schaal (1999). However, we don’t have sufficient information to infer the third gene flow event from Pampa to Atlantic forest ecoregion.

## Conclusion

In this study, we characterized genetic diversity and investigated population structure, performed duplicate analysis, estimated linkage disequilibrium, population differentiation and effective population size among the identified genetics groups in Brazilian cassava. Accurate identification of crop cultivars is crucial in assessing the impact of crop improvement program outputs. It also highlights the magnitude of genetic drift among identified genetic pools and its impact on survival, reproduction and the rate at which a population enters the extinction vortex. Minimizing loss of genetic variation via genetic drift should be an important objective of the cassava breeding program in Brazil. Based on our datasets, we suggest that: (1) there are 1536 IBS-selected unique accessions that represent the diversity within Brazilian germplasm collections, (2) there are 5 major ancestral populations and 10 genetic groups in the Brazilian germplasm collection studied, (3) selection based on traits (i.e., waxy, sugary and bitter related traits) of interest by breeders and farmers could have led to the observed population structure in the collected germplasm across Brazil, (4) spatial analysis showed with consistency the Southwestern Amazonia as the center of domestication of cassava, highlighting the cassava diversity landscape of Brazil, including impact of breeding activities, (5) LD decay across the genome indicates that interval of 1300–4700 SNP markers is required to detect with reasonable power, association of large quantitative trait loci (QTL) effect, based on our GBS dataset, (6) in addition, this defines the SNP density for development of PCR amplicon sequencing for genotyping and also suggests that GBS can be done at higher DNA multiplexing, thereby reducing the costs of variety fingerprinting, (7) we found evidence for *M. glaziovii* introgressions in Brazilian germplasm which may have resulted from past breeding activities, (8) the identification of *M. glaziovii* introgressions (and a possible third species sampled by UFB_002) in Brazil may indicate that interspecific hybridization is a more widespread phenomenon than previously suspected, and an effort to sequence and assemble the complete genomes of related *Manihot* species would enable a more comprehensive survey for sources of desirable alleles/traits and (9) finally, we suggest that cassava may have been propagated to the coastal region of Brazil independently of its migration from the Amazonian ecoregion. In a context where Amazonia’s in situ genetic resources are facing increasing conservation challenges, current findings present significant progress in characterizing ex situ cassava germplasm collections. Results include the identification of crop cultivars for efficient germplasm management, SNP density requirement for the development of simple PCR amplicon assay for genotyping. This study provides important information on the genome-wide assessments of the genetic landscape of cassava in Brazil as future resources for breeding activities, such as genomic selection, association studies and marker-assisted selection to increase genetic gain in cassava breeding programs in Brazil.

## Supplementary Information

Below is the link to the electronic supplementary material.

Supplementary file1 (XLSX 1889 kb)

Supplementary file2 (DOCX 1271 kb)

## Data Availability

Genotyping (SNP) data used in this study for 3,345 accessions were deposited on cassavabase.org hosted at “ftp://ftp.cassavabase.org/manuscripts/Ogbonna_et_al_2020/population_structure_manuscript”.

## References

[CR1] Akakpo R, Nora S, Hana C, Alexandre D, Gustave D, Anne-Céline T, Bénédicte R, Olivier F, Karine A, Vigouroux Y (2017). Molecular basis of African yam domestication: analyses of selection point to root development, starch biosynthesis, and photosynthesis related genes. BMC Genom.

[CR2] Albuquerque M (1969) A mandioca na Amazônia. Superintendência do Desenvolvimento de Amazônia

[CR3] Albuquerque HYG, Carmo CD, Brito AC, Oliveira EJ (2018). Genetic diversity of *Manihot esculenta* crantz germplasm based on single-nucleotide polymorphism markers. Annals Appl Biol.

[CR4] Alexander DH, November J, Lange K (2009). Fast model-based estimation of ancestry in unrelated individuals. Genome Res.

[CR5] Allem AC, Hillocks RJ, Thresh JM (2001). The origins and taxonomy of cassava. Cassava: biology, production and utilization.

[CR6] Allendorf FW (1986). Genetic drift and the loss of alleles versus heterozygosity. Zoo Biol.

[CR7] Alves-Pereira A, Clement CR, Picanço-Rodrigues D, Veasey EA, Dequigiovanni G, Ramos SLF, Pinheiro JB, Zucchi MI (2018). Patterns of nuclear and chloroplast genetic diversity and structure of manioc along major Brazilian Amazonian rivers. Ann Bot.

[CR8] Arroyo-Kalin M (2010). The Amazonian formative: crop domestication and anthropogenic soils. Diversity.

[CR9] Bauchet G, Grenier S, Samson N, Bonnet J, Grivet L, Causse M (2017). Use of modern tomato breeding germplasm for deciphering the genetic control of agronomical traits by genome wide association study. Theor Appl Genet.

[CR10] Biomas e sistema costeiro-marinho do Brasil in Coordenação de Recursos Naturais e Estudos Ambientais (2019) compatível com a escala 1:250 000, INSTITUTO BRASILEIRO DE GEOGRAFIA E ESTATÍSTICA (IBGE), p.112, ISBN: 9788524045103

[CR11] Botstein D, White RL, Skolnick M, Davis RW (1980). Construction of a genetic linkage map in man using restriction fragment length polymorphisms. Am J Hum Genet.

[CR12] Bradbury MG, Egan SV, Howard BJ (1999). Picrate paper kits for determination of total cyanogens in cassava roots and all forms of cyanogens in cassava products. J Sci Food Agri.

[CR13] Bredeson JV, Lyons JB, Prochnik SE, Wu GA, Ha CM, Edsinger-Gonzales E, Grimwood J (2016). Sequencing wild and cultivated cassava and related species reveals extensive interspecific hybridization and genetic diversity. Nat Biotechnol.

[CR14] Browning BL, Browning SR (2009). A unified approach to genotype imputation and haplotype-phase inference for large data sets of trios and unrelated individuals. Am J Hum Genet.

[CR15] Caye K, Deist TM, Martins H, Michel OH, François O (2016). TESS3: Fast inference of spatial population structure and genome scans for selection. Mol Ecol Res.

[CR16] Ceballos H, Iglesias CA, Pérez JC, Dixon AGO (2004). Cassava breeding: opportunities and challenges. Plant Mol Biol.

[CR17] Ceballos H, Pérez JC, Barandica OJ, Lenis JI, Morante N, Calle F, Pino L, Hershey CH (2016). Cassava breeding I: the value of breeding value. Front Plant Sci.

[CR18] Chang CC, Chow CC, Tellier LC, Vattikuti S, Purcell SM, Lee SJ (2015). Second-generation PLINK: rising to the challenge of larger and richer datasets. GigaScience.

[CR19] Clement C, Cristo-Araújo MD, D’Eeckenbrugge GC, Alves-Pereira A, Picanço-Rodrigues D (2010). Origin and domestication of native Amazonian crops. Diversity.

[CR20] Coates BS, Sumerford DV, Miller NJ, Kim KS, Sappington TW, Siegfried BD, Lewis LC (2009). Comparative performance of single nucleotide polymorphism and microsatellite markers for population genetic analysis. J Hered.

[CR101] Danecek P, Auton A, Abecasis G, Albers CA, Banks E, DePristo MA, Handsaker RE, Lunter G, Marth GT, Sherry ST, McVean G, Durbin R (2011). The variant call format and VCFtools. Bioinformatics.

[CR21] Do C, Waples RS, Peel D, Macbeth GM, Tillett BJ, Ovenden JR (2014). NeEstimator v2: re-implementation of software for the estimation of contemporary effective population size (Ne) from genetic data. Mol Ecol Res.

[CR22] Doyle JJ, Doyle JL (1987). A rapid DNA isolation procedure for small quantities of fresh leaf tissue. Phytochem Bull.

[CR23] Dwivedi SL, Ceccarelli S, Blair MW, Upadhyaya HD, Are AK, Ortiz R (2016). Landrace germplasm for improving yield and abiotic stress adaptation. Trends Plant Sci.

[CR24] Elias M, Mühlen GS, McKey D, Roa AC, Tohme J (2004). Genetic diversity of traditional South American landraces of cassava (*Manihot esculenta* Crantz): an analysis using microsatellites. Econ Bot.

[CR25] Elshire RJ, Glaubitz JC, Sun Q, Poland JA, Kawamoto K, Buckler ES (2011). A robust, simple genotyping-by-sequencing (GBS) approach for high diversity species. PLoS ONE.

[CR26] Eltaher S, Sallam A, Belamkar V, Emara HA, Nower AA, Salem KFM, Poland J, Baenziger PS (2018). Genetic diversity and population structure of F3:6 nebraska winter wheat genotypes using genotyping-by-sequencing. Front Genet.

[CR27] Eriksson D, Akoroda M, Azmach G, Labuschagne M, Mahungus N, Ortiz R (2018). Measuring the impact of plant breeding on sub-Saharan African staple crops. Outlook Agri.

[CR28] Ferguson ME, Shah T, Kulakow P, Ceballos H (2019). A global overview of cassava genetic diversity. PLoS ONE.

[CR29] Flint-Garcia SA, Thornsberry JM, Buckler ES (2003). Structure of linkage disequilibrium in plants. Annu Rev Plant Biol.

[CR30] François O, Durand E (2010). Spatially explicit bayesian clustering models in population genetics. Mol Ecol Res.

[CR31] Franklin IR, Frankham R (1998). How large must populations be to retain evolutionary potential?. Anim Conserv.

[CR32] Fraser JA, Alves-Pereira A, Junqueira AB, Peroni N, Clement CR (2012). Convergent adaptations: bitter manioc cultivation systems in fertile anthropogenic dark earths and floodplain soils in Central Amazonia. PLoS ONE.

[CR33] Frichot E, Mathieu F, Trouillon T, Bouchard G, François O (2014). Fast and efficient estimation of individual ancestry coefficients. Genetics.

[CR34] Glaubitz JC, Casstevens TM, Lu F, Harriman J, Elshire RJ (2014). TASSEL-GBS: A high capacity genotyping by sequencing analysis pipeline. PLoS ONE.

[CR36] Gosselin T (2019) radiator: RADseq data exploration, manipulation and visualization using R. 10.5281/zenodo.1475182. https://thierrygosselin.github.io/radiator/

[CR37] Greenbaum G, Templeton AR, Zarmi Y, Bar-David S (2014). Allelic richness following population founding events—a stochastic modeling framework incorporating gene flow and genetic drift. PLoS ONE.

[CR38] Jannink J (2010). Dynamics of long-term genomic selection. Genet Sel Evol.

[CR39] Johnson NL, Manyong VM, Dixon AGO, Pachico D, Evenson RE, Gollin D (2002). The impact of IARC genetic improvement programmes on cassava. Crop variety improvement and its effect on productivity: the impact of international agricultural research.

[CR40] Jombart T (2008). Adegenet: A R package for the multivariate analysis of genetic markers. Bioinformatics.

[CR41] Jombart T, Devillard S, Balloux F (2010). Discriminant analysis of principal components: a new method for the analysis of genetically structured populations. BMC Genet.

[CR42] Kawano K (2003). Thirty years of cassava breeding for productivity—biological and social factors for success. Crop Sci.

[CR102] Kilian B, Graner A (2012). NGS technologies for analyzing germplasm diversity in genebanks*. Brief Funct Genom.

[CR43] Kim S, Plagnol V, Hu T (2007). Recombination and linkage disequilibrium in arabidopsis thaliana. Nat Genet.

[CR44] Kittipadakul P, Kongsil P, Phumichai C, Hershey C (2017). Breeding cassava for higher yield. Achieving sustainable cultivation of cassava volume 2: genetics, breeding, pests and diseases.

[CR45] Kuon J, Qi W, Schläpfer P (2019). Haplotype-resolved genomes of geminivirus-resistant and geminivirus-susceptible African cassava cultivars. BMC Biol.

[CR46] Lefévre F, Charrier A (1993). Heredity of seventeen isozyme loci in cassava (*Manihot esculenta* Crantz). Euphytica.

[CR47] Léotard G, Duputié A, Kjellberg F (2009). Phylogeography and the origin of cassava: new insights from the northern rim of the Amazonian basin. Mol Phylogenet Evol.

[CR48] Lombardo U, Iriarte J, Hilbert L (2020). Early holocene crop cultivation and landscape modification in Amazonia. Nature.

[CR49] Luo Z, Brock J, Dyer JM, Kutchan T, Schachtman D, Augustin M, Ge Y, Fahlgren N, Abdel-Haleem H (2019). Genetic diversity and population structure of a camelina sativa spring panel. Front Plant Sci.

[CR50] Manel S, Joost S, Epperson BK, Holderegger R, Storfer A, Rosenberg MS, Scribner KT, Bonin A, Fortin M (2010). Perspectives on the use of landscape genetics to detect genetic adaptive variation in the field. Mol Ecol.

[CR51] McKenna A, Hanna M, Banks E (2010). The genome analysis toolkit: a mapreduce framework for analyzing next-generation DNA sequencing data. Genome Res.

[CR52] McKey D, Elias M, Pujol B, Duputié A (2010). The evolutionary ecology of clonally propagated domesticated plants. New Phytol.

[CR53] Milanesi M, Capomaccio S, Vajana E, Bomba L, Garcia JF, Ajmone-Marsan P, Colli L (2017) BITE: An R package for biodiversity analyses. bioRxiv. 10.1101/181610

[CR54] Miller Orson K, Singer R (1972). Flora neotropica; monograph no. 3, omphalinae (Clitocybeae-Tricholomataceae, Basidiomycetes). Mycologia.

[CR55] Miller A, Schaal B (2005). Domestication of a Mesoamerican cultivated fruit tree, spondias purpurea. Proc Natl Acad Sci USA.

[CR56] Mühlen GS, Alves-Pereira A, Carvalho CRL (2019). Genetic diversity and population structure show different patterns of diffusion for bitter and sweet manioc in Brazil. Genet Resour Crop Evol.

[CR57] Nassar NM, Hashimoto DY, Fernandes SD (2008). Wild manihot species: botanical aspects, geographic distribution and economic value. Genet Mol Res.

[CR58] Nei M (1987). Molecular evolutionary genetics.

[CR59] Nichols RFW (1947). Breeding cassava for virus resistance. East Afr Agri J.

[CR60] Niu S, Song Q, Koiwa H (2019). Genetic diversity, linkage disequilibrium, and population structure analysis of the tea plant (Camellia Sinensis) from an origin center, Guizhou Plateau, using genome-wide SNPs developed by genotyping-by-sequencing. BMC Plant Biol.

[CR61] Noli E, Teriaca MS, Conti S (2013). Criteria for the definition of similarity thresholds for identifying essentially derived varieties. Plant Breed.

[CR62] Nordborg M, Borevitz J, Bergelson J (2002). The extent of linkage disequilibrium in arabidopsis thaliana. Nat Genet.

[CR63] Nordenskiold E (1924) The ethnography of South‐America seen from Mojos in Bolivia. In: Comparative Ethnographical Studies 3. Oxford University Press, London. 10.1007/s11032-012-9773-0

[CR64] Ogbonna AC, de Andrade LGB, Rabbi IY, Mueller LA, Oliveira EJ, Bauchet GJ (2020). Large-scale GWAS using historical data identifies a conserved genetic architecture of cyanogenic glucosides content in cassava (*Manihot esculenta* Crantz) root. Plant J.

[CR65] Okechukwu RU, Dixon AGO (2008). Genetic gains from 30 years of cassava breeding in Nigeria for storage root yield and disease resistance in elite cassava genotypes. J Crop Improv.

[CR66] Oliveira EJ, Ferreira CF, da Silva SV (2014). Potential of SNP markers for the characterization of Brazilian cassava germplasm. Theor Appl Genet.

[CR67] Olsen KM (2004). SNPs, SSRs and inferences on cassava’s origin. Plant Mol Biol.

[CR68] Olsen KM, Schaal BA (1999). Evidence on the origin of cassava: phylogeography of *Manihot esculenta*. Proc Natl Acad Sci USA.

[CR69] Olsen K, Schaal B (2001). Microsatellite variation in cassava (*Manihot esculenta*, Euphorbiaceae) and its wild relatives: further evidence for a Southern Amazonian origin of domestication. Am J Bot.

[CR70] Paradis E (2011). Analysis of phylogenetics and evolution with R.

[CR71] Peprah BB, Parkes E, Manu-Aduening J, Kulakow P, van Biljon A, Labuschagne M (2020). Genetic variability, stability and heritability for quality and yield characteristics in provitamin a cassava varieties. Euphytica.

[CR72] Pickrell JK, Pritchard JK (2012). Inference of population splits and mixtures from genome-wide allele frequency data. PLoS Genet.

[CR73] Prochnik S, Marri PR, Desany B (2012). The cassava genome: current progress future directions. Trop Plant Biol.

[CR74] Purcell S, Neale B, Todd-Brown K, Thomas L, Ferreira MAR, Bender D, Maller J (2007). PLINK: A tool set for whole-genome association and population-based linkage analyses. Am J Hum Genet.

[CR75] R. Core Team (2015) An Introduction to R. Samurai Media Limited

[CR76] Rabbi IY, Hamblin MT, Kumar LP, Gedil MA, Ikpan AS, Jannink J, Kulakow PA (2014). High-resolution mapping of resistance to cassava mosaic geminiviruses in cassava using genotyping-by-sequencing and its implications for breeding. Virus Res.

[CR77] Rabbi IY, Kulakow PA, Manu-Aduening JA, Dankyi AA, Asibuo JY, Parkes EY, Abdoulaye T (2015). Tracking crop varieties using genotyping-by-sequencing markers: a case study using cassava (*Manihot esculenta* Crantz). BMC Genet.

[CR78] Rabbi IY, Udoh LI, Wolfe M, Parkes EY, Gedil MA, Dixon A, Ramu P, Jannink J, Kulakow P (2017). Genome-wide association mapping of correlated traits in cassava: dry matter and total carotenoid content. Plant Genome.

[CR79] Ramu P, Esuma W, Kawuki R, Rabbi IY, Egesi C, Bredeson JV, Bart RS, Verma J, Buckler ES, Lu F (2017). Cassava haplotype map highlights fixation of deleterious mutations during clonal propagation. Nat Genet.

[CR80] Razifard H, Ramos A, Della Valle AL, Bodary C (2020). Genomic evidence for complex domestication history of the cultivated tomato in Latin America. Mol Biol Evol.

[CR81] Renvoize BS (1972). The area of origin of *Manihot esculenta* as a crop plant—a review of the evidence. Econ Bot.

[CR82] Sardos J, Rouard M, Hueber Y, Cenci A, Hyma KE (2016). A genome-wide association study on the seedless phenotype in banana (Musa Spp.) reveals the potential of a selected panel to detect candidate genes in a vegetatively propagated crop. PLoS ONE.

[CR83] Schaal BA, Olsen KM, Luiz JC (2006). Evolution, domestication, and agrobiodiversity in the tropical crop cassava. Darwin’s Harvest.

[CR84] Sharma SK, MacKenzie K, McLean K, Dale F, Daniels S, Bryan GJ (2018). Linkage disequilibrium and evaluation of genome-wide association mapping models in tetraploid potato. G3.

[CR85] Sovic M, Fries A, Martin SA, Gibbs LH (2019). Genetic signatures of small effective population sizes and demographic declines in an endangered rattlesnake sistrurus catenatus. Evol Appl.

[CR86] Stich B, Urbany C, Hoffmann P, Gebhardt C (2013). Population structure and linkage disequilibrium in diploid and tetraploid potato revealed by genome-wide high-density genotyping using the SolCAP SNP array. Plant Breed.

[CR87] Van Inghelandt D, Reif JC, Dhillon BS, Flament P, Melchinger AE (2011). Extent and genome-wide distribution of linkage disequilibrium in commercial maize germplasm. Theor Appl Genet.

[CR88] Vos PG, Paulo MJ, Voorrips RE, Visser RGF, van Eck HJ, van Eeuwijk FA (2017). Evaluation of LD decay and various LD-decay estimators in simulated and SNP-array data of tetraploid potato. Theor Appl Genet.

[CR89] Wadl PA, Olukolu BA, Branham SE, Jarret RL, Yencho CG, Jackson MD (2018). Genetic diversity and population structure of the USDA sweetpotato (Ipomoea Batatas) germplasm collections using GBSpoly. Front Plant Sci.

[CR90] Walker RT, Simmons C, Arima E, Yi G-M, Antunes A, Waylen M, Irigaray M (2019). Avoiding Amazonian catastrophes: prospects for conservation in the 21st century. One Earth.

[CR91] Waples RS, Do C (2008). Ldne: a program for estimating effective population size from data on linkage disequilibrium. Mol Ecol Resour.

[CR92] Weir BS (1997). Genetic data analysis II. Biometrics.

[CR93] Weir BS, Cockerham CC (1984). Estimating F-statistics for the analysis of population structure. Int J Org Evol.

[CR94] Wolfe MD, Rabbi IY, Egesi C, Hamblin M, Kawuki R, Kulakow P (2016). Genome-wide association and prediction reveals genetic architecture of cassava mosaic disease resistance and prospects for rapid genetic improvement. Plant Genome.

[CR95] Wolfe MD, Bauchet GJ, Chan AW, Lozano R, Ramu P (2019). Historical introgressions from a wild relative of modern cassava improved important traits and may be under balancing selection. Genetics.

[CR96] Wolfgang S (2019). Selective sweeps. Genetics.

[CR97] Yabe S, Hara T, Ueno M, Enoki H, Kimura T (2018). Potential of genomic selection in mass selection breeding of an allogamous crop: an empirical study to increase yield of common buckwheat. Front Plant Sci.

[CR98] Yabe S, Iwata H, Jean-Luc Jannink J (2018). Impact of mislabeling on genomic selection in cassava breeding. Crop Sci.

[CR99] Yan J, Shah T, Warburton ML, Buckler ES, McMullen MD, Crouch J (2009). Genetic characterization and linkage disequilibrium estimation of a global maize collection using SNP markers. PLoS ONE.

[CR103] Yu J, Pressoir G, Briggs WH, Vroh Bi I, Yamasaki M, Doebley JF, McMullen MD, Gaut BS, Nielsen DM, Holland JB, Kresovich S, Buckler ES (2006). A unified mixed-model method for association mapping that accounts for multiple levels of relatedness. Nat Genet.

[CR100] Zhu X, Dong L, Jiang L, Li H, Sun L, Zhang H, Yu W (2015). Constructing a linkage–linkage disequilibrium map using dominant-segregating markers. DNA Res.

